# SGCE Promotes Breast Cancer Stem Cells by Stabilizing EGFR

**DOI:** 10.1002/advs.201903700

**Published:** 2020-06-08

**Authors:** Lina Zhao, Ting Qiu, Dewei Jiang, Haibo Xu, Li Zou, Qin Yang, Ceshi Chen, Baowei Jiao

**Affiliations:** ^1^ State Key Laboratory of Genetic Resources and Evolution Kunming Institute of Zoology Chinese Academy of Sciences Kunming Yunnan 650223 China; ^2^ Kunming College of Life Science University of Chinese Academy of Sciences Kunming Yunnan 650223 China; ^3^ Key Laboratory of Animal Models and Human Disease Mechanisms of the Chinese Academy of Sciences and Yunnan Province Kunming Institute of Zoology Chinese Academy of Sciences Kunming Yunnan 650223 China; ^4^ KIZ‐CUHK Joint Laboratory of Bioresources and Molecular Research in Common Diseases Kunming Institute of Zoology Chinese Academy of Sciences Kunming Yunnan 650223 China; ^5^ Center for Excellence in Animal Evolution and Genetics Chinese Academy of Sciences Kunming Yunnan 650223 China

**Keywords:** breast cancer stem cells, epidermal growth factor receptors, SGCE

## Abstract

Breast cancer stem cells (BCSCs) are responsible for resistance to chemotherapy, high degree of metastasis, and poor prognosis, especially in triple‐negative breast cancer (TNBC). The CD24^low^CD44^high^ and high aldehyde dehydrogenase 1 (ALDH1) cell subpopulation (CD24^low^CD44^high^ ALDH1^+^) exhibit very high tumor initiating capacity. In the current study, the upregulated genes are analyzed in both CD24^low^CD44^high^ and ALDH1^+^ cell populations at single‐cell resolution, and a highly expressed membrane protein, SGCE, is identified in both BCSC populations. Further results show that SGCE depletion reduces BCSC self‐renewal, chemoresistance, and metastasis both in vitro and in vivo, partially through affecting the accumulation of extracellular matrix (ECM). For the underlying mechanism, SGCE functions as a sponge molecule for the interaction between epidermal growth factor receptor (EGFR) and its E3 ubiquitination ligase (c‐Cbl), and thus inhibits EGFR lysosomal degradation to stabilize the EGFR protein. SGCE knockdown promotes sensitivity to EGFR tyrosine kinase inhibitors (TKIs), providing new clues for deciphering the current failure of targeting EGFR in clinical trials and highlighting a novel candidate for BCSC stemness regulation.

## Graphical abstract

SGCE is identified as a regulator of breast cancer stem cells (BCSCs) self‐renewal, chemosensitivity, and metastasis. SGCE loss increases degradation of epidermal growth factor receptor (EGFR) through enhanced interaction between EGFR and its ubiquitination ligase c‐Cbl, providing a potential mechanism for understanding the failure of EGFR‐targeted treatment in breast cancer.

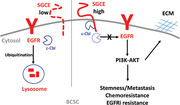

## 1 Introduction

Breast cancer is the most commonly diagnosed cancer worldwide and the leading cancer among women.^[^ ^1^ ^]^ Triple‐negative breast cancer (TNBC), which is negative for expression of estrogen receptor (ER*α*), progesterone receptor (PR), and human epidermal growth factor receptor (HER2), is the most aggressive form and carries a higher risk of metastasis than any other breast cancer subtype.^[^ ^2^ ^]^ Due to the lack of the above receptors, TNBC does not respond to hormonal or anti‐HER2 therapies, with treatment usually based on traditional chemotherapy, although poly (ADP‐ribose) polymerase (PARP) inhibitors and anti‐programmed death‐ligand 1 (PD‐L1) monoclonal antibodies (mAbs) have been approved for a subset of TNBC patients.^[^ ^2^ ^]^ Breast cancer stem cells (BCSCs) are a small subpopulation of self‐renewing cancer cells responsible for drug resistance, cancer initiation, and cancer progression.^[^ ^3^, ^4^ ^]^ Several breast cancer cell subpopulations, including cells with CD24^low^CD44^high^ expression ^[^ ^4^ ^]^ and cells with high aldehyde dehydrogenase 1 (ALDH1) activity,^[^ ^5^ ^]^ are considered as BCSCs because they initiate tumor growth. Although chemotherapy can kill tumor cells in TNBC, it fails to eliminate BCSCs, which may be the reason for recurrence and drug resistance.^[^ ^6^, ^7^ ^]^

Epidermal growth factor receptor (EGFR), which belongs to the receptor tyrosine kinase family,^[^ ^8^ ^]^ is important for drug resistance, cancer stem cells, and metastasis in different types of cancer.^[^ ^9^ ^]^ EGFR expression in solid tumors, including breast cancer, is 20–50‐fold higher than that reported in normal tissues,^[^ ^10^ ^]^ and high EGFR expression is found in 69% of TNBC.^[^ ^11^ ^]^ Therefore, treatment targeting EGFR, including inhibitors of EGFR (e.g., tyrosine kinase inhibitors, TKIs) and mAbs have been taken efforts. Gefitinib, a small‐molecule kinase inhibitor that directly competes with adenosine triphosphate (ATP) to bind to EGFR, can inhibit EGFR autophosphorylation and downstream signaling.^[^ ^12^, ^13^ ^]^ Although such inhibitors have been used with success in nonsmall cell lung cancer (NSCLC) treatment, clinical trials in breast cancer have shown poor results, even in combination with chemotherapy.^[^ ^14^, ^15^ ^]^ It has been reported that NSCLC‐resistant cells enhance EGFR levels due to dysregulated degradation following loss of binding to its E3 ubiquitin ligase Cbl.^[^ ^16^ ^]^ In addition, it has been hypothesized that mAb and TKI resistance are partly due to activation of alternative downstream pathways of EGFR.

SGCE is the *ε* isoform member of the sarcoglycan family and includes transmembrane components in a dystrophin–glycoprotein complex. This complex helps to stabilize muscle fiber membranes and links the muscle cytoskeleton to the extracellular matrix (ECM). Although other sarcoglycan family members are primarily expressed in muscle, SGCE is expressed broadly.^[^ ^17^, ^18^ ^]^ Furthermore, mutations in SGCE are related to myoclonus‐dystonia syndrome (MDS) and inherited movement disorder.^[^ ^19^ ^]^ Although correlation studies have shown that SGCE is involved in colorectal ^[^ ^20^ ^]^ and gastric cancers,^[^ ^20^, ^21^ ^]^ how it participates in cancer remains elusive. In the present study, we found SGCE to be highly expressed in BCSCs and closely related with BCSC self‐renewal, tumorigenesis, and chemoresistance as well as ECM deposition and remodeling. Moreover, SGCE was involved in anti‐EGFR resistance, which is implicated in targeted therapy for TNBC, thus providing new clues for deciphering the current failure of EGFR targeted therapies in clinical trials.

## 2 Results

### 2.1 SGCE is Highly Enriched in BCSCs

**Figure 1:**
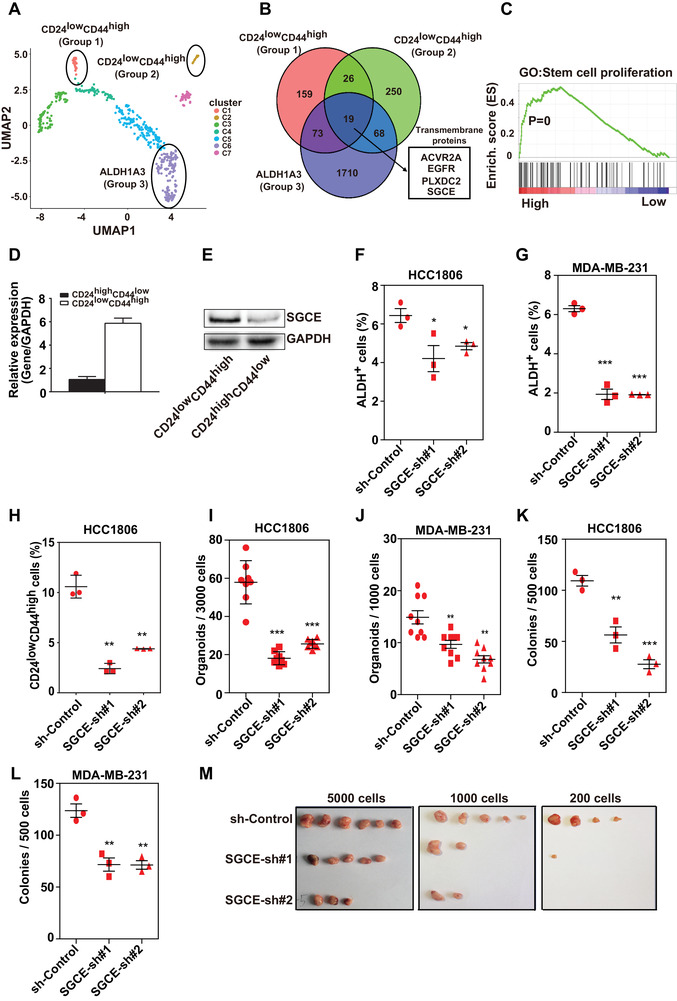
Loss of SGCE inhibits stemness of BCSCs. A) UMAP plot showing distribution of stemness‐related groups of TNBC epithelial cells (groups are separated by expression of CD24, CD44, and ALDH1A3). B) Venn diagram showing intersection of highly expressed genes between each stemness‐related group and nonstemness‐related group. C) GSEA showing enrichment of stem cell proliferation in SGCE differentially expressed genes based on TCGA. D) mRNA and E) protein expression levels of SGCE in BCSC populations. ALDH analysis upon SGCE knockdown in F) HCC1806 and G) MDA‐MB‐231 cells. H) Assay of CD24^low^CD44^high^ population upon SGCE knockdown in HCC1806 cells. Tumorsphere assay upon SGCE knockdown in I) HCC1806 and J) MDA‐MB‐231 cells. Clonal formation assay upon SGCE knockdown in K) HCC1806 and L) MDA‐MB‐231 cells. M) Xenograft assay using CD24^low^CD44^high^ fraction upon SGCE knockdown in HCC1806 cells.

BCSC populations are well recognized using markers of CD24^low^CD44^high^ and ALDH^+^. In the current study, we analyzed the expression profile in TNBC at single‐cell resolution based on published single‐cell RNA‐Seq data.^[^ ^22^ ^]^ Our analysis showed that CD24^low^CD44^high^ cells were clustered into two populations (group 1 and group 2). Furthermore, cells with high expression of ALDH, which is predominantly composed of the ALDH1A3 isoform,^[^ ^5^ ^]^ were also clustered together (group 3) (**Figure** **1**A and Figure S1A–C, Supporting Information). As the overlapping population (CD24^low^CD44^high^ALDH^+^ cells) exhibits very high tumor initiating capacity,^[^ ^23^ ^]^ we analyzed the upregulated genes in the above three groups and found 19 highly and commonly expressed genes (Figure 1B). Among these genes, we identified four membrane‐bound genes (Figure 1B and Figure S1D–G, Supporting Information), which are critical for signal transduction, cell function, and targeting.^[^ ^24^ ^]^ ACVR2A, a bone morphogenetic protein (BMP) receptor, is reported to be a tumor suppressor,^[^ ^25^, ^26^ ^]^ and PLXDC2, a novel mitogen for neural progenitors, is associated with paclitaxel resistance.^[^ ^27^, ^28^ ^]^ Moreover, the expression of *SGCE* was positively correlated with the highly expressed genes identified in the CD24^low^CD44^high^ALDH^+^ populations based on breast cancer patient data obtained from the Cancer Genome Atlas (TCGA) database (Table S1, Supporting Information).^[^ ^29^, ^30^ ^]^ We therefore selected the *SGCE* gene for further study.

Based on the TCGA breast cancer dataset, gene set enrichment analysis (GSEA) revealed strong correlations between stem cell proliferation gene category and samples with high SGCE expression (Figure 1C). The breast cancer stem‐like subpopulation (i.e., CD24^low^CD44^high^) was isolated from breast cancer cells (HCC1806). We found that SGCE was highly expressed in the BCSC subpopulation (CD24^low^CD44^high^) compared with the non‐BCSC subpopulation (CD24^high^CD44^low^) at both the mRNA and protein level (Figure 1D,E). Moreover, TNBC patients with high SGCE levels showed poor prognosis in both relapse‐free survival and distal metastasis‐free survival analyses (Figure S2A,B, Supporting Information). We also found SGCE to be more highly expressed in TNBC than in other subtypes of breast cancer (Figure S2C, Supporting Information).

### 2.2 Knockdown of SGCE Reduces Stemness of BCSCs

To investigate whether SGCE is a functional gene in BCSCs, we examined cancer stemness properties using flow cytometry analysis of BCSC markers as well as suspension tumorsphere and clonal formation assays in breast cancer cells. Based on well‐recognized BCSC markers,^[^ ^4^, ^5^ ^]^ the ratios of both the ALDH^+^ (Figure 1F,G) and CD24^low^CD44^high^ (Figure 1H) cell fractions clearly decreased upon SGCE knockdown in TNBC cells (HCC1806 and MDA‐MB‐231). As MDA‐MB‐231 cells are almost all positive for CD44 staining,^[^ ^31^ ^]^ we checked CD24^low^CD44^high^ populations in HCC1937 cells, and found the same pattern (Figure S2D, Supporting Information). The knockdown efficiencies by short hairpin RNA (shRNA) in the above three cell lines are shown in Figure S2E in the Supporting Information. Overexpression of SGCE increased the ratios of both ALDH^+^ (Figure S3A,B, Supporting Information) and CD24^low^CD44^high^ (Figure S3C, Supporting Information) in TNBC cells. The overexpression efficiencies are shown in Figure S2F in the Supporting Information. Knockdown of SGCE also remarkably reduced the number of tumorsphere and the ability of clonal formation in both HCC1806 and MDA‐MB‐231 cells (Figure 1I–L). Overexpression of SGCE increased tumorsphere number and clonal formation ability in both TNBC cell lines (Figure S3D–G, Supporting Information). Transplantations of HCC1806 cells with limiting dilution revealed lower frequencies of tumor formation, even in a small number of cells upon SGCE knockdown (**Table** **1**). Furthermore, limiting dilution assay using the CD24^low^CD44^high^ population showed that the tumor formation probability was severely reduced upon SGCE knockdown (Figure 1M, **Table** **2**). The control group generated tumors with as few as 200 cells, whereas the SGCE‐knockdown group did not form any tumors. Taken together, our data suggest that SGCE is closely related with tumor initiation both in vitro and in vivo.

**Table 1:** Statistical results of xenograft assay upon SGCE knockdown using whole HCC1806 cell line

Cell number	sh‐Control	SGCE‐sh#1	SGCE‐sh#2
4 × 10^6^ cells	8/8	8/8	6/8
4 × 10^5^ cells	10/10	7/8	5/8
4 × 10^4^ cells	6/8	5/8	1/8
TIC frequency	1/288 541 (1/68 570–1/12 142)	1/10 8421 (1/255 433–1/46 021)	1/1 463 997 (1/3 201 790–1/669 403)
*P* value		*P* < 0.05	*P* < 0.01

**Table 2:** Statistical results of xenograft assay using CD24^low^CD44^high^ fraction upon SGCE knockdown

Cell number	sh‐Control	SGCE‐sh#1	SGCE‐sh#2
5000 cells	6/8	5/8	3/8
1000 cells	5/8	2/8	2/8
200 cells	4/8	1/8	0/8
TIC frequency	1/1656 (1/3287–1/835)	1/4192 (1/8783–1/2001)	1/8062 (1/19 893–1/3268)
*P* value		*P* < 0.05	*P* < 0.01

### 2.3 Loss of SGCE Increases Chemotherapy Sensitivity of TNBC through BCSCs

**Figure 2:**
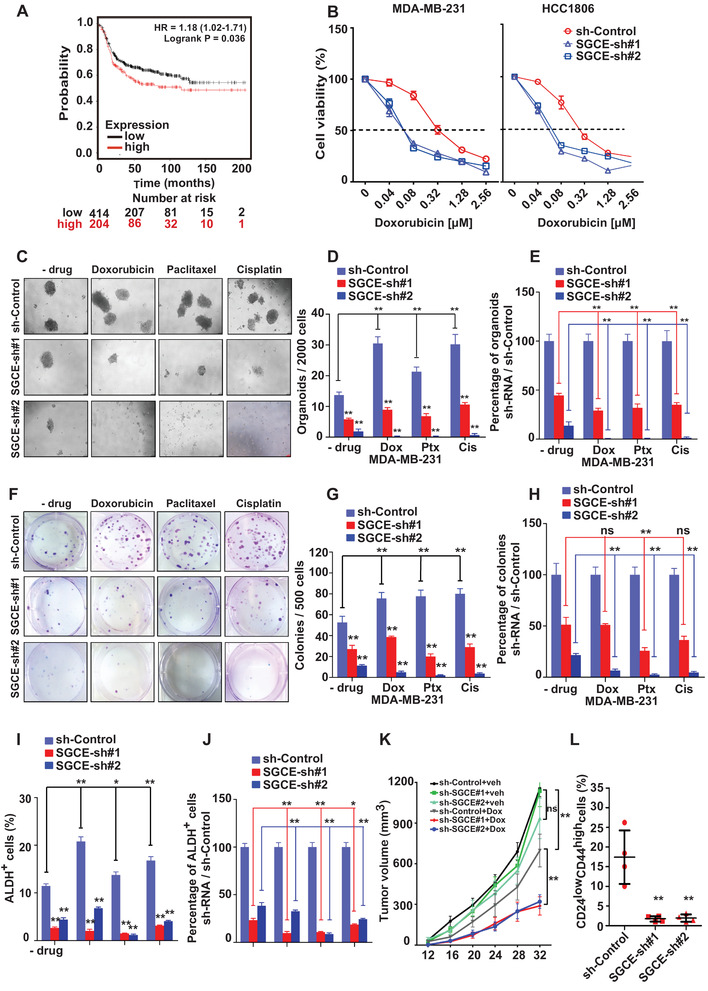
Loss of SGCE increases chemotherapy sensitivity of breast cancer through BCSCs. A) Kaplan–Meier survival analysis showing correlation between relapse‐free survival (RFS) and SGCE expression in breast cancer patients exposed to chemotherapy. B) Cell viability after treatment with different concentrations of doxorubicin in HCC1806 and MDA‐MB‐231 cells (*n* = 6). C) Tumorsphere and F) clonal formation assays, and their number and percentage calculations D,E) for (C), G,H) for (F) following treatment with doxorubicin, paclitaxel, and cisplatin in SGCE‐depleted MDA‐MB‐231 cells. FACS analysis of I) CD24^low^CD44^high^ population and J) percentage calculations following treatment with doxorubicin, paclitaxel, and cisplatin in SGCE‐depleted MDA‐MB‐231 cells. K) Tumor growth curve following SGCE knockdown in HCC1806 cells with or without doxorubicin. L) FACS analysis of CD24^low^CD44^high^ population in SGCE‐knockdown xenograft assay with doxorubicin. (*) *P* < 0.05; (**) *P* < 0.01. Dox: doxorubicin; Ptx: paclitaxel; Cis: cisplatin.

As failure to eliminate cancer stem cells greatly contributes to drug resistance,^[^ ^32^, ^33^ ^]^ we investigated whether SGCE played a role in chemotherapy sensitivity. Using survival data of patients exposed to chemotherapy, Kaplan–Meir plots showed that patients with lower SGCE expression levels were correlated with better prognosis (**Figure** **2**A). In our sensitivity assay using TNBC cells, SGCE‐knockdown cells showed a much lower cell viability dosage (50% inhibitory concentration, IC50) to chemotherapy (doxorubicin in Figure 2B and cisplatin in Figure S3H,I, Supporting Information). To confirm that the observed effects were due to BCSCs, we investigated stem‐like activities following treatment with doxorubicin, cisplatin, and paclitaxel using an in vitro sphere‐culture system and clonal formation assay. Drug treatments significantly increased the numbers of colonies and spheres (first rows in Figure 2C–F, light blue columns in Figure 2D,G Figure S3J,K, Supporting Information), suggesting enrichment of BCSCs after chemotherapy, consistent with previous reports.^[^ ^34^, ^35^ ^]^ This enrichment was significantly reduced following SGCE knockdown (Figure 2C,D,F,G and Figure S3J,K, Supporting Information). Flow cytometry analysis also indicated that enrichment of the ALDH^+^ and CD24^low^CD44^high^ populations was abrogated after SGCE interruption with drug treatment (Figure 2I and Figure S3L,M, Supporting Information). The combined effects of shRNA and drug treatment showed significant differences compared to drug treatment only in the above assays (Figure 2E,H,J, and Figure S3N–Q, Supporting Information), suggesting reduced resistance to drugs upon SGCE knockdown. We next treated the xenograft tumors with doxorubicin. While SGCE silencing alone did not lead to a significant decrease in tumor volume, under treatment, SGCE depletion significantly reduced tumor growth (Figure 2K), suggesting an obvious improvement in the therapeutic effects of doxorubicin when SGCE expression levels are low. Among the tumors, the BCSC fractions of CD24^low^CD44^high^ significantly decreased following SGCE knockdown (Figure 2L). Thus, the above data indicate that SGCE loss increases chemotherapy sensitivity of TNBC through BCSCs.

### 2.4 SGCE Promotes ECM Deposition through Cancer‐Associated Fibroblasts (CAFs)

**Figure 3:**
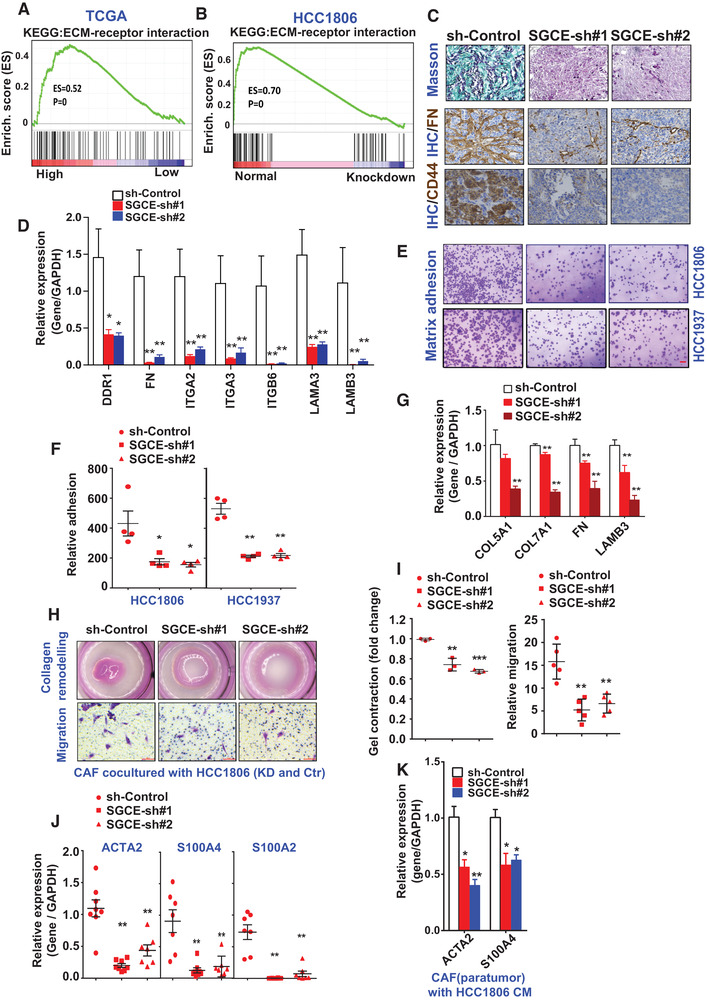
SGCE promotes ECM deposition. GSEA showing enrichment of ECM‐receptor interaction in SGCE differentially expressed genes based on A) TCGA and B) RNA‐Seq data. C) Masson (up) and immunohistochemical staining (bottom) of FN and CD44 in xenograft tumors. D) mRNA levels of ECM‐related genes in SGCE‐knockdown xenograft tumors. E,F) Matrix adhesion assay in SGCE‐depleted cell lines. G) mRNA levels of ECM‐related genes in CAFs co‐cultured with SGCE‐knockdown HCC1806 cells. H,I) Collagen remodeling and migration abilities of TNBC CAFs co‐cultured with HCC1806 cells upon SGCE knockdown. J) mRNA levels of CAF markers in xenograft tumors. K) mRNA levels of CAF‐related genes in TNBC CAFs treated with conditional medium from SGCE‐depleted HCC1806 cells. (*) *P* < 0.05; (**) *P* < 0.01.

Previous studies have reported that ECM, including collagens, fibronectin (FN), laminins, proteoglycans, and matricellular proteins, is involved in stemness maintenance in BCSCs.^[^ ^36^ ^]^ Therefore, we investigated the correlation between SGCE and ECM. Using the TCGA database, we carried out GSEA based on SGCE expression. Results revealed that ECM‐receptor interaction was among the top KEGG pathways (Figure S4D, Supporting Information) and was positively related to SGCE expression levels in breast cancer (**Figure** **3**A). In the co‐expression network based on expression levels, SGCE was in the top hub position in TNBC (Figure S4A, Supporting Information), suggesting a crucial role of SGCE within ECM components. To further confirm the involvement of SGCE in regulating ECM, SGCE‐knockdown cells were used for RNA‐sequencing. The GSEA results revealed that ECM‐receptor interaction enrichment was reduced upon SGCE knockdown in HCC1806 cells (Figure 3B). The top GO terms for the differentially expressed genes (DEGs) after SGCE knockdown were related to ECM‐receptor interaction and cell adhesion (Figure S4B,C, Supporting Information), further suggesting the involvement of SGCE in ECM‐receptor interaction.

Masson trichrome staining for collagen detection was applied to determine the expression levels of ECM components. Results indicated that collagen was remarkably reduced (Figure 3C) upon SGCE knockdown in the xenografted tumor tissues developed in Table 1, and the other ECM components, including fibronectin 1, and representative receptors, including CD44, were also decreased upon SGCE knockdown (Figure 3C). The reduction of ECM and receptor genes was confirmed by quantitative real‐time polymerase chain reaction (q‐RT‐PCR) (Figure 3D). Under collagen coating, matrix adhesion to collagen in the HCC1806 and HCC1937 cells was significantly reduced upon SGCE knockdown (Figure 3E,F), implying the loss of interaction between ECM and its receptors.

As CAF cells can deposit ECM components and facilitate cancer development,^[^ ^37^ ^]^ we next investigated whether CAF cells were regulated by SGCE. When CAF cells derived from patient breast tumors were co‐cultured with conditional medium of SGCE‐knockdown HCC1806 cells, CAF exhibited lower ECM expression (Figure 3G) and stiffness (Figure 3H, upper, and 3I, left). To observe the recruitment ability of cancer cells for CAFs in vivo, we detected CAF markers in SGCE knocked‐down tumors. Results showed a remarkable reduction in the expression levels of CAF markers, including ACTA2, S100A4, and S100A2 (Figure 3J). Our data clearly demonstrated a reduction in the migration ability of CAFs (Figure 3H, lower, and Figure 3I, right), with the expression of markers of activated fibroblasts also decreased (Figure 3K), suggesting that without SGCE, migration and transformation abilities were severely downregulated.

### 2.5 SGCE Regulates BCSCs and ECM through EGFR

**Figure 4:**
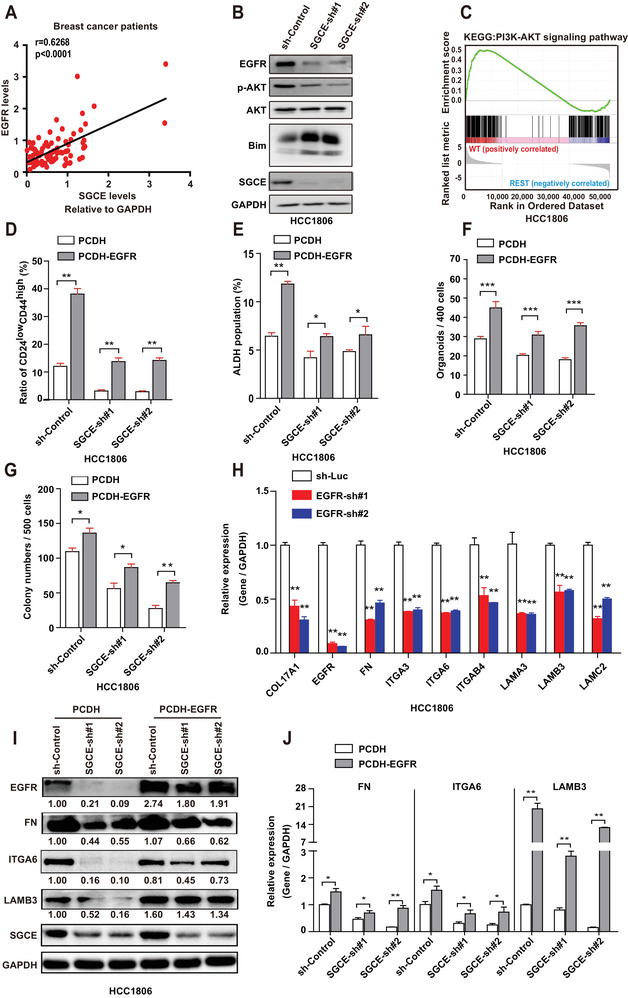
SGCE regulates BCSCs and ECM through EGFR. A) Correlation analysis of SGCE and EGFR in breast cancer patients by immunoblotting analysis. B) Western blot of EGFR and related genes in SGCE‐knockdown HCC1806 cells. C) KEGG enrichment analysis of PI3K‐AKT signaling pathway in SGCE‐knockdown HCC1806 cells. Ratio of D) CD24^low^CD44^high^ and E) ALDH^+^ populations in SGCE‐knockdown HCC1806 cells with EGFR overexpression. F) Number of tumorspheres and G) colony number in SGCE‐knockdown HCC1806 cells with EGFR overexpression. H) mRNA levels of ECM‐related genes in EGFR knockdown cells. I) Protein and J) mRNA levels in ECM‐related genes in SGCE‐depleted‐EGFR overexpressed HCC1806 cells. (*) *P* < 0.05; (**) *P* < 0.01; (***) *P* < 0.001.

To explore how SGCE maintains stemness of BCSCs, we determined the expression correlations among SGCE and other proteins involved in stemness. In our collected patient samples, EGFR levels were positively correlated with SGCE levels (**Figure** **4**A). EGFR is considered crucial in promoting the stemness of BCSCs ^[^ ^38^ ^]^ and in the accumulation of ECM.^[^ ^11^ ^]^ We therefore checked whether the expression levels of EGFR were affected by lower expression of SGCE. In TNBC cells (HCC1806 and MDA‐MB‐231), EGFR protein levels were downregulated with loss of SGCE (Figure 4B and Figure S5A, Supporting Information). The PI3K‐AKT signaling pathway, which is downstream of EGFR, was also downregulated in SGCE‐knockdown HCC1806 cells based on KEGG analysis of RNA‐Seq data (Figure 4C). In accordance with the expression levels, the downstream signal of EGFR, i.e., p‐AKT, was de‐activated upon SGCE knockdown (Figure 4B and Figure S5A, Supporting Information). We also examined the PI3K‐AKT downstream molecule, Bim.^[^ ^39^ ^]^ Results showed that Bim was upregulated upon SGCE knockdown, suggesting the involvement of PI3K‐AKT signaling in SGCE effects. We performed a series of assays to further confirm whether the effects of SGCE were mediated by EGFR signaling. Results showed that the BCSC phenotypes (including CD24^low^CD44^high^ and ALDH^+^) as well as tumorsphere number and clonal formation were partially restored when EGFR was overexpressed upon SGCE knockdown (Figure 4D–G and Figure S5B–D, Supporting Information). To explore whether EGFR could influence ECM, we carried out EGFR interference and found that ECM‐related genes were significantly downregulated in both HCC1806 and MDA‐MB‐231 cells (Figure 4H and Figure S5E, Supporting Information). In addition, we found that the reduction in ECM genes following SGCE knockdown could be partially rescued by EGFR overexpression in both HCC1806 and MDA‐MB‐231 cells (Figure 4I,J and Figure S5F, Supporting Information). Thus, the above data suggest that SGCE regulates BCSCs and ECM through EGFR.

### 2.6 SGCE Knockdown Results in EGFR Degradation through Lysosomes

**Figure 5:**
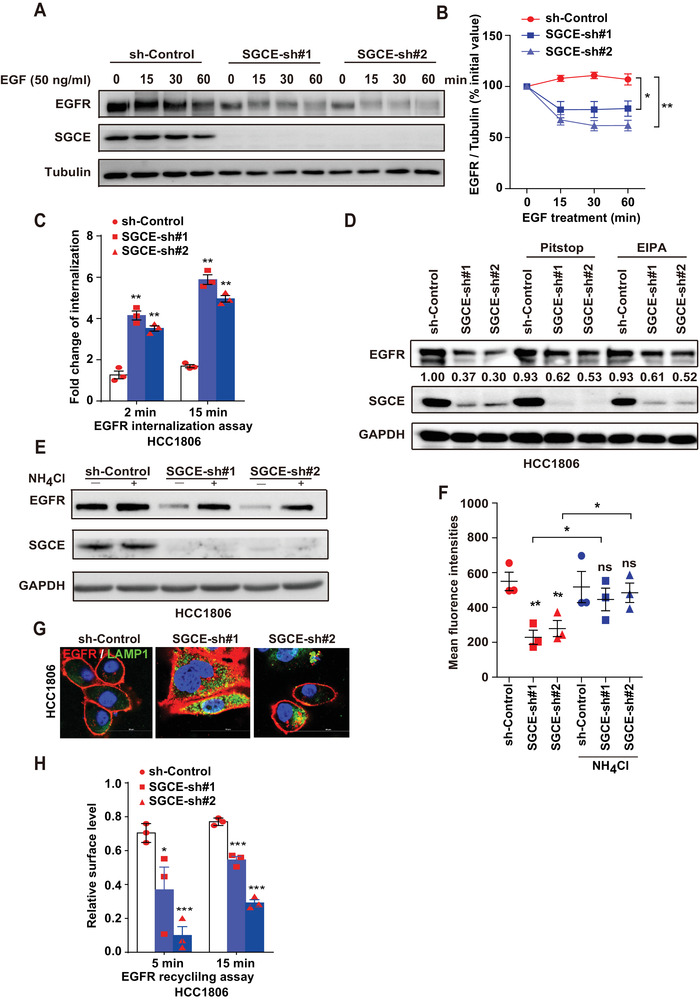
SGCE regulates EGFR lysosomal degradation. A) SGCE‐knockdown HCC1806 cells were incubated in serum‐deprived medium overnight and stimulated with 50 ng mL^−1^ human EGF for indicated time points, followed by whole‐cell lysate extraction for immunoblotting analysis with indicated antibodies. B) Quantifications of relative expression of EGFR in (A). C) Fold change of EGFR internalization at different time points upon SGCE knockdown. D) Immunoblotting analysis of EGFR in SGCE‐knockdown HCC1806 cells with internalization inhibitors. E) Immunoblotting analysis of EGFR in SGCE‐knockdown HCC1806 cells along with NH_4_Cl treatment. F) Detection of membrane‐bound EGFR using FACS in SGCE‐knockdown HCC1806 cells with NH_4_Cl treatment. G) Co‐localization of EGFR and LAMP1 in HCC1806 cells. H) For EGFR recycling assay, amount recycled 5 and 15 min after initial EGF stimulation was quantified and EGFR recycling ratio was calculated [MFI (*T*) – MFI (*T*pulse)/MFI (T0) – MFI (*T*pulse)] × 100. (*) *P* < 0.05; (**) *P* < 0.01; (***) *P* < 0.001.

To investigate the dynamic changes in EGFR, we treated cells with EGF. Results showed that EGFR was highly expressed up to 30 min, which was due to the stimulation effects by EGF, consistent with previous reports.^[^ ^40^ ^]^ In contrast, EGFR rapidly decreased at the same time points after EGF exposure in SGCE‐knockdown cells (**Figure** **5**A,B), suggesting that SGCE loss can lead to degradation of EGFR. As receptor internalization is a necessary step for EGFR degradation,^[^ ^41^ ^]^ we determined the internalized EGFR levels upon SGCE knockdown. Results demonstrated that EGFR internalization was significantly promoted at both time points observed (Figure 5C). To further confirm the above phenomenon, we investigated how SGCE affected signaling of EGFR internalization. EGFR internalization can occur via multiple ways, such as macropinocytosis and micropinocytosis, including clathrin‐mediated endocytosis (CME) and nonclathrin endocytosis (NCE).^[^ ^42^ ^]^ We investigated EGFR levels by blocking CME and macropinocytosis by their specific inhibitors, Pitstop and EIPA, respectively.^[^ ^43^, ^44^ ^]^ Upon treatment, Pitstop and EIPA partially recovered the reduction in EGFR protein caused by SGCE knockdown in both HCC1806 and MDA‐MB‐231 cells (Figure 5D and Figure S6A, Supporting Information). These data suggest that SGCE depletion promotes EGFR internalization and degradation through both the CME and macropinocytosis pathways.

To investigate how SGCE loss resulted in EGFR degradation, we treated cells with NH_4_Cl to inhibit lysosomal activity. Results showed that NH_4_Cl treatment recovered EGFR protein levels (Figure 5E and Figure S6B, Supporting Information) upon SGCE knockdown. In the fluorescence activated cell sorting (FACS) assay using EGFR‐conjugated antibody, we also found that EGFR levels were significantly rescued by NH_4_Cl treatment upon SGCE knockdown (Figure 5F and Figure S6C, Supporting Information). To confirm whether EGFR was degraded in lysosomes, we observed the co‐localization of EGFR and lysosome marker (LAMP1). Results showed that EGFR and LAMP1 exhibited preferential enrichment of EGFR in the lysosomal compartment of SGCE‐knockdown cells (Figure 5G and Figure S6D, Supporting Information). Moreover, considering that EGFR can also be degraded by proteasomes, we treated cells with proteasome inhibitor (MG132) and found that EGFR could not be rescued (Figure S6E,F, Supporting Information), thus suggesting that SGCE does not influence EGFR degradation through proteasomal activity. After its internalization, EGFR faces two fates:, i.e., degradation by lysosomes or recycling to the cell membrane.^[^ ^45^ ^]^ Our results showed a markedly reduced recycling rate (Figure 5H), suggesting that SGCE knockdown led to greater EGFR lysosomal degradation and reduced EGFR recycling to the membrane.

### 2.7 SGCE Knockdown Promotes Interaction between EGFR and c‐Cbl

**Figure 6:**
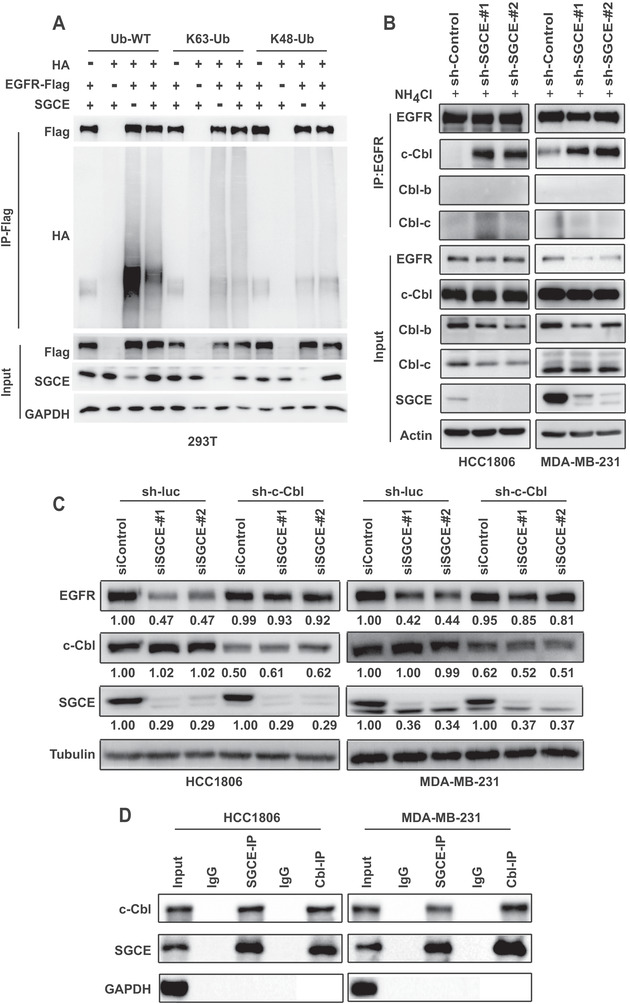
SGCE regulates EGFR degradation by enhancing interaction of EGFR and Cbl. A) Ubiquitination of Flag‐EGFR when co‐transfected with HA‐Ub and SGCE expression plasmids in 293T cells. B) Co‐immunoprecipitation of EGFR and c‐Cbl, Cbl‐b, and Cbl‐c with NH_4_Cl treatment in SGCE‐depleted HCC1806 and MDA‐MB‐231 cells. C) Immunoblotting analysis of EGFR along with c‐Cbl interference in SGCE‐depleted HCC1806 and MDA‐MB‐231 cells. sh‐luc is scramble control for sh‐c‐Cbl. D) Co‐immunoprecipitation of SGCE and c‐Cbl in HCC1806 and MDA‐MB‐231 cells in vivo. (*) *P* < 0.05; (**) *P* < 0.01; (***) *P* < 0.001.

As ubiquitination is necessary for EGFR degradation, we attempted to establish the regulatory correlation between SGCE and EGFR ubiquitination. Overexpression of SGCE inhibited total EGFR ubiquitination and K63‐linked ubiquitination, which directed to lysosomal degradation, but not K48‐linked ubiquitination, thus resulting in proteasomal degradation (**Figure** **6**A). This further confirmed that EGFR degradation caused by SGCE knockdown was mediated through lysosomal degradation. As SGCE did not interact directly with EGFR (Figure S6G, Supporting Information), we speculated that SGCE affected EGFR ubiquitination via other molecules. The E3 RING ligase Cbl, which contains c‐Cbl, Cbl‐b, and Cbl‐c, can directly bind to EGFR.^[^ ^46^ ^]^ Furthermore, GRB2, as an adaptor protein ^[^ ^47^, ^48^ ^]^ between Cbl and EGFR, can cause EGFR ubiquitination and lysosomal degradation.^[^ ^49^, ^50^, ^51^ ^]^ Thus, we investigated whether SGCE loss changed the interaction between EGFR and the Cbl family or GRB2. Results demonstrated obviously enhanced EGFR binding to c‐Cbl, but not to Cbl‐b or Cbl‐c upon SGCE knockdown, whereas the binding was undetected or very weak at normal SGCE expression levels (Figure 6B). We did not observe enhanced interaction between EGFR and GRB2 (Figure S6H, Supporting Information). To confirm the role of c‐Cbl in mediating EGFR degradation by SGCE, we investigated whether c‐Cbl could rescue its downregulation. Results showed that degradation of EGFR was abolished when c‐Cbl was knocked down along with SGCE depletion (Figure 6C). To demonstrate the interaction between SGCE and c‐Cbl, a co‐immunoprecipitation pull‐down (co‐IP) assay was carried out, which showed a clear interaction between SGCE and c‐Cbl at the endogenous level (Figure 6D).

### 2.8 SGCE Promotes Metastasis of Breast Cancer Cells and Drug Resistance‐Targeted EGFR

**Figure 7:**
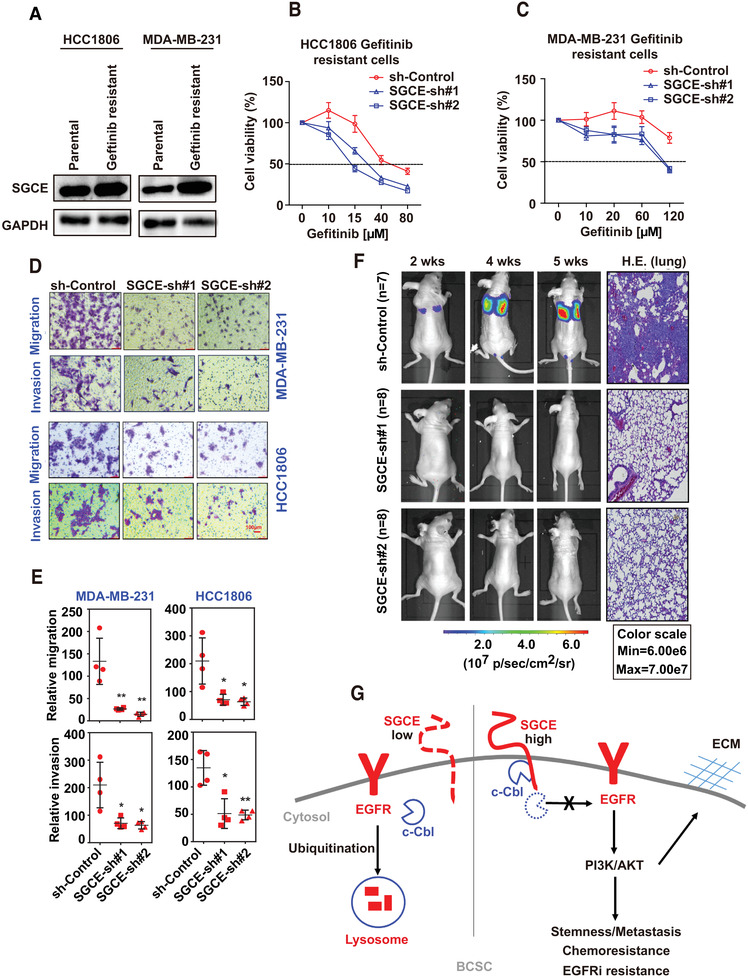
SGCE promotes metastasis of breast cancer cells and drug resistance‐targeted EGFR. A) Immunoblotting analysis of SGCE in gefitinib‐resistant and parental cell lines. Drug sensitivity experiment following SGCE knockdown in B) gefitinib‐resistant HCC1806 and C) MDA‐MB‐231 cell lines. D,E) Migration and invasion abilities of HCC1806 and MDA‐MB‐231 cells following SGCE knockdown and statistical results. F) Luciferase signal intensities in mice after tail‐vein injection with MDA‐MB231 cells expressing indicated constructs and HE‐staining of lungs. G) Working model: In ALDH^+^CD24^low^CD44^high^ BCSCs, SGCE is highly expressed to maintain stemness, metastasis, chemotherapy, and EGFRi resistance. When SGCE is knocked down, the interaction between SGCE and c‐Cbl is disturbed, and c‐Cbl is free to bind to EGFR, leading to EGFR lysosomal degradation and downregulation of AKT pathway as well as ECM deposition and remodeling. These effects make breast cancer cells sensitive to chemotherapy and EGFRi. (*) *P* < 0.05; (**) *P* < 0.01.

Upregulation of the AKT, c‐Met, and NF‐κB pathways can contribute to cell survival and resistance to TKIs.^[^ ^52^, ^53^, ^54^ ^]^ In our study, GSEA revealed that the PI3K‐AKT and NK‐kB pathways were downregulated in HCC1806 cells following SGCE knockdown (Figure 4C and Figure S7A, Supporting Information). SGCE deletion attenuated AKT, Ikk*α*, and IkB*α* phosphorylation as well as c‐Met expression (Figure 4B and Figures S5A, S7B–E, Supporting Information). The above results suggest that SGCE may be involved in EGFR TKI resistance. Gefitinib, a small‐molecule EGFR kinase inhibitor successfully used in nonsmall lung cancer treatment, is not beneficial for breast cancer patients.^[^ ^55^ ^]^ We generated gefitinib‐resistant cells (Figure S7F,G, Supporting Information) and found that SGCE was highly upregulated in the gefitinib‐resistant HCC1806 and MDA‐MB‐231 cell lines compared to the parental cell lines (**Figure** **7**A). Therefore, we examined whether SGCE knockdown could influence the effects of gefitinib. As shown, sensitivity to gefitinib increased in SGCE‐depleted cells, which substantially reduced the maximum IC50 required in gefitinib‐resistant HCC1806 and MDA‐MB‐231 cell lines (Figure 7B,C). Moreover, using the same approach, we tested another EGFR inhibitor, lapatinib, in resistant cells (Figure S7H,I, Supporting Information), with results demonstrating lower IC50 upon SGCE knockdown (Figure S7J,K, Supporting Information).

As tumor metastasis is the readout of cancer stem cells, we examined metastasis following SGCE knockdown. In both TNBC cell lines, SGCE knockdown significantly attenuated cell migration and invasion abilities (Figure 7D,E). To confirm metastasis ability, we injected MDA‐MB‐231 expressing sh‐SGCE together with the luciferase gene into the tail vein of mice. Compared with the control group, the mice that received a tail‐vein injection of SGCE‐knockdown cells developed fewer lung metastases (Figure 7F). These data preliminarily suggest that knockdown of SGCE inhibits metastasis of TNBC.

## 3 Discussion

The expression of EGFR is 20–50‐fold higher in solid tumors, including breast cancer, compared to that found in normal tissues.^[^ ^10^ ^]^ Moreover, EGFR is overexpressed in more than 50% of TNBC patients, which is markedly higher than that for other subtypes of breast cancer ^[^ ^56^ ^]^ and is significantly correlated with poor prognosis.^[^ ^57^ ^]^ Therefore, drug treatments targeting EGFR, including inhibitors of EGFR (TKIs) and mAbs, have been developed over the last two decades. However, clinical trials targeting EGFR have shown poor benefits for breast cancer patients.^[^ ^55^ ^]^ This could be partially due to deregulated EGFR degradation and reduced ubiquitination as well as enhanced EGFR recycling in EGFR‐TKI resistant cells.^[^ ^58^ ^]^ In our results, SGCE loss increased EGFR ubiquitination and lysosomal degradation and reduced EGFR recycling, implying that SGCE may play an important role in EGFR‐TKI resistance. It has been reported that cetuximab‐resistant cells have increased EGFR levels due to reduced degradation from loss of binding to Cbl.^[^ ^16^ ^]^ Our study revealed that SGCE interacted with c‐Cbl, and SGCE knockdown promoted EGFR degradation by increasing the interaction between EGFR and c‐Cbl (Figure 7G). Furthermore, SGCE knockdown promoted drug sensitivity in gefitinib‐ and lapatinib‐ resistant TNBC cell lines (Figure 7 and Figure S7, Supporting Information), which may be related to the high expression of SGCE in such cells (Figure 7A). The upregulation of the AKT, c‐Met, and NF‐*κ*B pathways is known to contribute to cell survival and resistance to TKIs.^[^ ^45^, ^46^, ^47^ ^]^ This agrees with our findings, which demonstrated that SGCE knockdown downregulated the PI3K/AKT, c‐Met, and NF‐*κ*B pathways. Thus, these results suggest that SGCE knockdown may increase drug sensitivity for EGFR in TNBC, which is important for clinical trials.

To investigate how SGCE regulates stemness of BCSCs, we explored the related mechanism by screening various signaling pathways using luciferase reporter constructs, including WNT, TGF*β*, RhoA, and oxidative stress. Most showed inconsistent regulatory effects by SGCE (Figure S8A, Supporting Information, data not shown), and only EGFR found to be consistently downregulated by SGCE knockdown. The rescue assays in Figure 4 showed that the effects of SGCE knockdown could be recovered by EGFR overexpression. Thus, we focused on EGFR as a downstream target of SGCE. Of note, the rescue assays did not completely recover stemness activities, implying other downstream mediators may be involved in the SGCE effects.

EGFR is well recognized for its regulatory roles in cancer cell proliferation and survival. On the other hand, the EGFR pathway is known to mediate BCSCs.^[^ ^59^, ^60^ ^]^ In clinical samples and patient‐derived xenografted models (PDXs), EGFR is highly correlated with stemness of breast cancer.^[^ ^61^, ^62^ ^]^ Furthermore, EGFR inhibitors are reported to be responsible for elimination of BCSCs.^[^ ^3^ ^]^ For downstream signaling, EGFR modulates BCSCs through both the PI3K‐AKT and MEK/ERK pathways in TNBC.^[^ ^63^, ^64^ ^]^ Besides PI3K‐AKT signaling (Figure 4), our data indicated that ERK phosphorylation decreased upon SGCE knockdown (Figure S8B, Supporting Information), further supporting the above reports. For downstream stemness‐related genes, we examined several well‐known genes, including Oct4, Nanog, and Sox2, which showed mild or inconsistent downregulation in the HCC1806 and MDA‐MB‐231 cell lines (Figure S8C, Supporting Information). Actually, many reports have demonstrated that BCSCs can be regulated independently of the above genes.^[^ ^65^, ^66^, ^67^, ^68^ ^]^

As an important component of the tumor microenvironment, ECM is a highly dynamic and complex network of surrounding cells. Interactions between ECM and tumor cells can impact cellular signaling pathways and are responsible for the effects of cancer therapy.^[^ ^69^, ^70^ ^]^ Based on bioinformatics analysis and experiments, we showed that SGCE regulated ECM deposition and remodeling through EGFR (Figure 3). Considering that ECM is closely related to cancer stem cell self‐renewal, tumor formation, and drug resistance,^[^ ^70^, ^71^, ^72^ ^]^ we speculate that the role of SGCE in regulating BCSCs and drug resistance may be through ECM. However, how SGCE influences ECM through EGFR needs further investigation. Our in vitro data on the maintenance of BCSC stemness (Figure 1) indicated that SGCE regulates ECM through paracrine, implying that SGCE regulates ECM independent of tumor niches, whereas the co‐culture assays showed that CAFs are involved in the promotion of BCSC stemness. As EGFR can regulate cancer stem cells through both intracellular signaling ^[^ ^5^ ^]^ and extracellular niches,^[^ ^59^, ^73^ ^]^ SGCE may exert its roles in multiple ways.

The first step of EGFR signaling regulation is its internalization. Both CME and several NCE pathways are involved in EGFR internalization.^[^ ^74^ ^]^ The ubiquitination of EGFR is necessary for all internalization pathways, and the ubiquitin ligase responsible for EGFR ubiquitination is Cbl, a ring‐finger domain E3 ubiquitin ligase.^[^ ^50^ ^]^ Cbl can directly bind to EGFR, or indirectly bind to EGFR via the adaptor protein Grb2.^[^ ^48^, ^74^ ^]^ The EGFR‐Grb2‐Cbl complex is necessary for NCE, with previous studies showing that EGFR mutants lacking Grb2 binding sites are completely defective for NCE.^[^ ^75^, ^76^ ^]^ Our results indicated that SGCE knockdown caused induction of EGFR internalization, and increased the interaction between EGFR and c‐Cbl but not that between EGFR and Grb2. Thus, we concluded that SGCE depletion did not regulate EGFR internalization through the NCE pathway. Moreover, the clathrin inhibitor (Pitstop) partially rescued EGFR protein level upon SGCE knockdown, suggesting that SGCE regulates EGFR internalization through the CME pathway.

Earlier research demonstrated that EGFR predominantly enters the recycling pathway through CME under stimulation of EGF at any concentration, but degrades via NCE,^[^ ^77^ ^]^ indicating that different internalization pathways lead to distinct EGFR fates (i.e., degradation versus recycling). However, upon SGCE knockdown, EGFR predominantly showed a degradation fate through CME (Figure 5), seemingly inconsistent with the above report. Actually, the levels of ubiquitination for EGFR are the critical threshold for EGFR fate.^[^ ^75^, ^76^ ^]^ We found that SGCE loss led to release of c‐Cbl and promotion of EGFR ubiquitination and thereafter EGFR degradation, implying ubiquitination may be the main factor for EGFR fate. Thus, our findings suggest the necessity of SGCE in EGFR fate, and provide further potential links among EGFR ubiquitination, degradation, and internalization pathways.

In summary, we identified SGCE as a new TNBC BCSC maintaining protein which stabilizes EGFR through sequestering its E3 ligase c‐Cbl. Depletion of SGCE suppresses TNBC cell stemness, proliferation, adhesion, migration, invasion, and drug resistance in vitro and metastasis in vivo. We propose that SGCE could serve as a diagnosis and prognosis biomarker and a therapeutic target for TNBC. Development of the targeting strategies against SGCE is warranted.

## 4 Experimental Section

##### Bioinformatics Analysis of scRNA‐Seq Data in TNBC

The TNBC single‐cell RNA‐Seq (scRNA‐Seq) data from a previous study ^[^ ^22^ ^]^ were obtained from the github website (https://github.com/Michorlab/tnbc_scrnaseq). Cell type assessment was retrieved from the original article. Tumor epithelial cells from three patients (PT039, PT089, PT081) were used for downstream analysis as those patients retained relatively higher epithelial cells after quality control (QC), which was performed using scater script in *R* (total_counts ≥ 1000, 10 000 ≥ total_features_by_counts ≥ 500, pct_counts_Mt ≤ 20).^[^ ^78^ ^]^ Dimensionality reduction and clustering analysis of epithelial cells were conducted using Monocle3 with default parameters.^[^ ^79^ ^]^ The expression levels of the CD24, CD44, and ALDH1A3 genes were used to retrieve the CD24^low^CD44^high^ and ALDH1A3^+^ stem cell clusters. The DEGs between each BCSC cluster and non‐BCSC cluster were determined using the “pairwiseWilcox” function in scran script (parameters: FDR ≤ 0.05, direction = “up”).^[^ ^80^ ^]^ The raw sequence data reported in this paper were deposited in the Genome Sequence Archive in the BIG Data Center, Beijing Institute of Genomics (BIG), Chinese Academy of Sciences, under accession number CRA002079, which is publicly accessible at http://bigd.big.ac.cn/gsa.

##### Cell Culture

Human breast cancer cell lines (HCC1806 and HCC1937) were grown in RPMI 1640 medium with 10% fetal bovine serum (FBS). The MDA‐MB‐231 cells were grown in Dulbecco's Modified Eagle Medium (DMEM)/F12 medium with 10% FBS. The 293T cells were cultured in DMEM medium supplemented with 10% FBS.

##### Mammosphere Assay

The HCC1806 and MDA‐MB‐231 cells were plated in ultralow attachment 96‐well plates with EpiCult‐B Basal Medium (Human) and Epicult‐B Proliferation Supplement (Human) with hydrocortisone and heparin. The mammosphere was calculated after 10–14 d.

##### Flow Cytometry

Cells were grown in 6‐well plates (10^5^ cells per well). After 48 h, cells were digested, counted, and stained with antibodies: anti‐CD44‐FITC (BD, 1:100) and anti‐CD24‐PE (BD, 1:100). ALDH enzymatic activity was assessed using an ALDEFLUOR kit (Promega) according to the provided manual. For the detection of EGFR on the membrane, when the cells had grown to 80% confluence, NH_4_Cl was added into the medium for 4 h and the cells were trypsinized. Cells were then blocked with 5% goat serum for 10 min. Cells were first stained with EGFR (Abcam, ab30) for 60 min on ice and then washed with phosphate‐buffered saline (PBS) followed by incubation with Alexa Fluor 555 secondary antibody (Life Technologies, 1:1000) on ice for 30 min.

##### Migration and Invasion Assay

After cells grew exponentially, they were seeded into 24‐well culture inserts with 8 µm pores. After 24 h, a cotton swab was applied to clean the cells on the upper surface of the filters. Cells on the lower filter surface were defined as the invaded cells. For easier visualization, the cells were fixed in 4% paraformaldehyde (PFA) and stained with 0.2% crystal violet. In the invasion assay, before seeding the cells, the inserts were first coated with Matrigel (BD) for 24 h, after which the cells and Matrigel were removed with a cotton swab.

##### Lung Metastasis

Luciferase‐expressing MDA‐MB‐231 cells with shSGCE and sh‐Control were injected into the tail veins of 8‐week‐old NOD/SCID mice. For bioluminescence imaging, mice were intraperitoneally injected with 100 mg g^−1^ of d‐luciferin in PBS. 5 min after injection, mice were anesthetized by anesthetic ketamine, and bioluminescence was imaged with a CCD camera. Five weeks later, all mice were euthanized by cervical dislocation for lung tissue sampling. All animal care and handling procedures were performed per the protocols approved by the Ethics Committee of the Kunming Institute of Zoology, Chinese Academy of Sciences (Permit No. SMKX‐20180306‐17).

##### Xenograft Assay

Nude female mice (aged 8 weeks) were used for studying tumorigenesis ability. The HCC1806 cells suspended in a 1:1 mixture of PBS and Matrigel (total volume of 100 µL) were injected into the mammary fat pads. After 8 weeks of adaptation, the presence of palpable tumors was examined. The number of tumor‐initiating cells was calculated using the extreme limiting dilution analysis (ELDA) web interface (http://bioinf.wehi.edu.au/software/elda/).

##### Quantitative RT‐PCR

Total RNA from cells and tumor tissues was extracted using Trizol based on the manufacturer's instructions. RNA was resuspended in RNase free water. Complementary DNA (cDNA) was produced from 1 µg of RNA using the ExScript RT Reagent Kit per the relevant instructions. The cDNA samples were subjected to q‐RT‐PCR using SYBR mix.

##### Clonal formation Assay

The HCC1806 and MDA‐MB‐231 cells were seeded into 6‐well plates at a density of 500 cells per well. Cells were cultured for 10 d, with the medium changed every 3 d. The cells were then fixed in 4% PFA and stained with 0.2% crystal violet.

##### shRNA Transduction

Cell lines that stably expressed the specific shRNA or nontargeted control shRNA (sh‐control) were constructed using the PLKO.1‐based lentiviral shRNA technique. The shRNA plasmid, along with PMD2.G and psPAX2 (4:1:3), was transfected into the 293T cells to produce lentiviral particles. The HCC1806 and MDA‐MB‐231 cells were infected with lentiviruses expressing the shRNA constructs with polybrene after cells were seeded into 6‐well plates for 16–24 h. Fresh medium was added after 24 h, with the cells then selected with puromycin to obtain cell lines stably expressing shRNA.

##### EGFR Recycling Assay

Cells with 80% confluence were starved for 8 h and collected, with one sample group defined as T0. Other samples were incubated with EGF for 1 h at 4 °C and collected, defined as the pulse group. The remaining samples were incubated at 37 °C for 15 min to allow internalization. Cells were treated with mild acid/salt to remove the bounded EGF and were chased for different periods at 37 °C to allow recycling. Cells were fixed in 1% formaldehyde at every time point. EGFR on the cell membrane was labeled using EGFR antibody and analyzed by FACS. The relative surface level of EGFR at each time point (*t*) was calculated from the mean fluorescence intensities (MFI) with the formula [MFI (*t*) – MFI (pulse)/MFI (T0) – MFI (pulse)] × 100.

##### EGFR Internalization Assay

EGFR internalization was assayed by flow cytometry according to previous report.^[^ ^40^ ^]^ Cells were seeded into 6‐well plates with complete medium and were starved without FBS for 6 h when confluence reached 80%. The cells were then treated with 10 × 10^−9^ m Alexa Fluor 555‐labeled EGF (Invitrogen) at 4 °C for 30 min. After washing and incubation for 2 min and internalization for 15 min, the cells were placed on ice to stop internalization and washed three times with cold PBS. The cells were then treated with an acid wash (0.2 m acetic acid and 0.5 m NaCl, pH 2.8), followed by washing with PBS to remove noninternalized EGF. Cells were washed and suspended in FACS buffer (2% FBS and 0.01% sodium azide in PBS) and fixed by adding an equal volume of 4% formaldehyde/PBS. The fluorescence emission of internalized EGF was detected by flow cytometry.

##### Generation of EGFRi‐Resistant Cell Line

EGFR‐TKI‐resistant sublines were established by stepwise escalation^[^ ^81^ ^]^ HCC1806 and MDA‐MB‐231 parental cells were cultured with stepwise escalation of concentrations of gefitinib (APExBIO) and lapatinib (Selleck) from 1 × 10^−6^ to 28 × 10^−6^ m over three months.

##### Cell Viability Assay

Cellular responses to treatments (i.e., doxorubicin, paclitaxel, cisplatin, gefitinib, and lapatinib) were estimated by cell viability assays. Cells were seeded in 96‐well plates at a concentration of 8000 cells per well with complete medium overnight and then treated with the respective reagents for two days. Cell viability was then measured by 3‐(4,5‐dimethylthiazol‐2‐yl)‐5‐(3‐carboxymethoxyphenyl)‐2‐(4‐sulfophenyl)‐2H‐tetrazolium (MTS) assay using the CellTiter 96 AQueous One Solution Reagent (Promega) at 490 nm.

##### Immunohistochemistry

For immunohistochemical analysis, paraffin sections were deparaffinized and rehydrated in a series of degraded alcohols. Antigen retrieval was performed using 10 × 10^−3^ m citrate buffer in a microwave for 20 min. Sections were incubated in 3% H_2_O_2_ for 15 min to inactivate endogenous peroxidase. The samples were blocked with 10% goat serum for 2 h and incubated with primary antibody FN (Sigma, 1:100) overnight at 4 °C followed by secondary antibody conjugated horseradish peroxidase (HRP, Sigma, 1:200) for 1 h. Finally, the slides were stained with 3,3‐diaminobenzidine (DAB) and countered with hematoxylin.

##### Immunofluorescence

The HCC1806 and MDA‐MB‐231 cells were seeded on coverslips in 4‐well plates (50 000 cells per well) and fixed in 4% PFA. Cells were then blocked with 0.1% TritonX‐100 in 5% goat serum for 1 h, followed by incubation with primary antibody EGFR (Abcam, 1:100) and LAMP1 (Abcam, 1:100) overnight at 4 °C. Fluorescein‐labeled (KPL, 1:200) and Alexa Fluor 555‐labeled secondary antibodies (Life Technologies, 1:2000) were used to treat cells for 1 h. Lastly, coverslips were mounted with 4′,6‐diamidino‐2‐phenylindole (DAPI) (Vector Laboratories) and observed via laser‐scanning confocal microscopy (Nikon).

##### Co‐IP and Western Blotting

Cells were washed with PBS and lysed in ice‐cold lysis buffer for 30 min with protease inhibitor (B14001, BioTools). Cell lysates were incubated with the indicated antibodies overnight at 4 °C, followed by incubation with protein A/G beads (Santa Cruz) for 3 h at 4 °C. The beads were washed with cell lysis buffer three to five times. Finally, the beads were boiled in 2 × SDS for 10 min. The eluents were analyzed by Western blotting. Lysis samples were separated by Sodium dodecylsulphate polyacrylamide gel electrophoresis (SDS‐PAGE), transferred onto polyvinylidene fluoride (PVDF) membranes, blocked with 5% nonfat dried milk for 1 h, incubated with indicated primary antibody at 4 °C overnight and with secondary antibody conjugated HRP for 1 h, and then detected with a chemiluminescent HRP substrate (Millipore).

## Conflict of Interest

The authors declare no conflict of interest.

## Author Contributions

L.Z., T.Q., and D.J. contributed equally to this work. L.Z., T.Q., C.C., and B.J. designed the experiments, interpreted the results, and wrote the manuscript. L.Z. and T.Q. performed the experiments. H.X. performed the bioinformatics analysis. D.J., Q.Y., and L. Zou provided experimental assistance.

## Supporting information

Supporting Information

## References

[1] F. Bray , J. Ferlay , I. Soerjomataram , R. L. Siegel , L. A. Torre , A. Jemal , Ca‐Cancer J. Clin. 2018, 68, 394. 30207593 10.3322/caac.21492

[2] A. Lee , M. B. A. Djamgoz , Cancer Treat. Rev. 2018, 62, 110. 29202431 10.1016/j.ctrv.2017.11.003

[3] X. Li , M. T. Lewis , J. Huang , C. Gutierrez , C. K. Osborne , M. F. Wu , S. G. Hilsenbeck , A. Pavlick , X. Zhang , G. C. Chamness , H. Wong , J. Rosen , J. C. Chang , JNCI, J. Natl. Cancer Inst. 2008, 100, 672. 18445819 10.1093/jnci/djn123

[4] M. Al‐Hajj , M. S. Wicha , A. Benito‐Hernandez , S. J. Morrison , M. F. Clarke , Proc. Natl. Acad. Sci. USA 2003, 100, 3983. 12629218 10.1073/pnas.0530291100 PMC153034

[5] C. Ginestier , M. H. Hur , E. Charafe‐Jauffret , F. Monville , J. Dutcher , M. Brown , J. Jacquemier , P. Viens , C. G. Kleer , S. Liu , A. Schott , D. Hayes , D. Birnbaum , M. S. Wicha , G. Dontu , Cell Stem Cell 2007, 1, 555. 18371393 10.1016/j.stem.2007.08.014 PMC2423808

[6] M. Bartucci , R. Dattilo , C. Moriconi , A. Pagliuca , M. Mottolese , G. Federici , A. D. Benedetto , M. Todaro , G. Stassi , F. Sperati , M. I. Amabile , E. Pilozzi , M. Patrizii , M. Biffoni , M. Maugeri‐Sacca , S. Piccolo , R. De Maria , Oncogene 2015, 34, 681. 24531710 10.1038/onc.2014.5

[7] S. Palomeras , S. Ruiz‐Martinez , T. Puig , Molecules 2018, 23, 2193. 10.3390/molecules23092193 PMC6225226 30200262

[8] N. E. Hynes , G. MacDonald , Curr. Opin. Cell Biol. 2009, 21, 177. 19208461 10.1016/j.ceb.2008.12.010

[9] K. K. Hampton , R. J. Craven , Oncoscience 2014, 1, 504. 10.18632/oncoscience.67 PMC4278327 25594057

[10] I. O. Alanazi , Z. Khan , Asian Pac. J. Cancer Prev. 2016, 17, 445. 26925626 10.7314/apjcp.2016.17.2.445

[11] Z. Koledova , X. Zhang , C. Streuli , R. B. Clarke , O. D. Klein , Z. Werb , P. Lu , Proc. Natl. Acad. Sci. USA 2016, 113, E5731. 27621461 10.1073/pnas.1611532113 PMC5047180

[12] C. L. Arteaga , Exp. Cell Res. 2003, 284, 122. 12648471 10.1016/s0014-4827(02)00104-0

[13] R. S. Herbst , M. Fukuoka , J. Baselga , Nat. Rev. Cancer 2004, 4, 956. 15573117 10.1038/nrc1506

[14] M. Bernsdorf , C. Ingvar , L. Jorgensen , M. K. Tuxen , E. H. Jakobsen , A. Saetersdal , M. L. Kimper‐Karl , N. Kroman , E. Balslev , B. Ejlertsen , Breast Cancer Res. Treat. 2011, 126, 463. 21234672 10.1007/s10549-011-1352-2

[15] A. Agrawal , E. Gutteridge , J. M. Gee , R. I. Nicholson , J. F. Robertson , Endocr.‐Relat. Cancer 2005, 12, S135. 16113090 10.1677/erc.1.01059

[16] D. L. Wheeler , S. Huang , T. J. Kruser , M. M. Nechrebecki , E. A. Armstrong , S. Benavente , V. Gondi , K. T. Hsu , P. M. Harari , Oncogene 2008, 27, 3944. 18297114 10.1038/onc.2008.19 PMC2903615

[17] V. Nigro , E. de Sá Moreira , G. Piluso , M. Vainzof , A. Belsito , L. Politano , A. A. Puca , M. R. Passos‐Bueno , M. Zatz , Nat. Genet. 1996, 14, 195. 8841194 10.1038/ng1096-195

[18] M. Imamura , K. Araishi , S. Noguchi , E. Ozawa , Hum. Mol. Genet. 2000, 9, 3091. 11115854 10.1093/hmg/9.20.3091

[19] N. Nardocci , G. Zorzi , C. Barzaghi , F. Zibordi , C. Ciano , D. Ghezzi , B. Garavaglia , Mov. Disord. 2008, 23, 28. 17853490 10.1002/mds.21715

[20] P. Ortega , A. Moran , T. Fernandez‐Marcelo , C. De Juan , C. Frias , J. A. Lopez‐Asenjo , A. Sanchez‐Pernaute , A. Torres , E. Diaz‐Rubio , P. Iniesta , M. Benito , Int. J. Oncol. 2010, 36, 1209. 20372795 10.3892/ijo_00000604

[21] J. L. Sepulveda , J. L. Gutierrez‐Pajares , A. Luna , Y. Yao , J. W. Tobias , S. Thomas , Y. Woo , F. Giorgi , E. V. Komissarova , A. Califano , T. C. Wang , A. R. Sepulveda , Mod. Pathol. 2016, 29, 182. 26769141 10.1038/modpathol.2015.144

[22] M. Karaayvaz , S. Cristea , S. M. Gillespie , A. P. Patel , R. Mylvaganam , C. C. Luo , M. C. Specht , B. E. Bernstein , F. Michor , L. W. Ellisen , Nat. Commun. 2018, 9, 3588. 30181541 10.1038/s41467-018-06052-0 PMC6123496

[23] S. Liu , Y. Cong , D. Wang , Y. Sun , L. Deng , Y. Liu , R. Martin‐Trevino , L. Shang , S. P. McDermott , M. D. Landis , S. Hong , A. Adams , R. D'Angelo , C. Ginestier , E. Charafe‐Jauffret , S. G. Clouthier , D. Birnbaum , S. T. Wong , M. Zhan , J. C. Chang , M. S. Wicha , Stem Cell Rep. 2014, 2, 78. 10.1016/j.stemcr.2013.11.009 PMC3916760 24511467

[24] K. R. Kampen , J. Membr. Biol. 2011, 242, 69. 21732009 10.1007/s00232-011-9381-7

[25] E. L. Alarmo , T. Kuukasjarvi , R. Karhu , A. Kallioniemi , Breast Cancer Res. Treat. 2007, 103, 239. 17004110 10.1007/s10549-006-9362-1

[26] D. Wodzinski , A. Wosiak , J. Pietrzak , R. Swiechowski , A. Jelen , E. Balcerczak , Genet. Mol. Biol. 2019, 42, 32. 30856244 10.1590/1678-4685-GMB-2017-0332 PMC6428132

[27] Y. Wang , H. Li , Oncol. Lett. 2018, 15, 9793. 29928353 10.3892/ol.2018.8600 PMC6004651

[28] S. F. Miller‐Delaney , I. Lieberam , P. Murphy , K. J. Mitchell , PLoS One 2011, 6, e14565. 21283688 10.1371/journal.pone.0014565 PMC3024984

[29] S. V. Vasaikar , P. Straub , J. Wang , B. Zhang , Nucleic Acids Res. 2018, 46, D956. 29136207 10.1093/nar/gkx1090 PMC5753188

[30] M. Liu , Y. Liu , L. Deng , D. Wang , X. He , L. Zhou , M. S. Wicha , F. Bai , S. Liu , Mol. Cancer 2018, 17, 65. 29471829 10.1186/s12943-018-0809-x PMC5824475

[31] C. Zhang , H. Gao , C. Li , J. Tu , Z. Chen , W. Su , X. Geng , X. Chen , J. Wang , W. Pan , Technol. Cancer Res. Treat. 2018, 17, 1. 10.1177/1533033818764497 PMC5909851 29658391

[32] E. Batlle , H. Clevers , Nat. Med. 2017, 23, 1124. 28985214 10.1038/nm.4409

[33] C. M. Fillmore , C. Kuperwasser , Breast Cancer Res. 2008, 10, R25. 18366788 10.1186/bcr1982 PMC2397524

[34] H. Lu , D. Samanta , L. Xiang , H. Zhang , H. Hu , I. Chen , J. W. Bullen , G. L. Semenza , Proc. Natl. Acad. Sci. USA 2015, 112, E4600. 26229077 10.1073/pnas.1513433112 PMC4547233

[35] H. Lu , L. Tran , Y. Park , I. Chen , J. Lan , Y. Xie , G. L. Semenza , Cancer Res. 2018, 78, 4191. 29880481 10.1158/0008-5472.CAN-18-0270

[36] C. Chang , H. L. Goel , H. Gao , B. Pursell , L. D. Shultz , D. L. Greiner , S. Ingerpuu , M. Patarroyo , S. Cao , E. Lim , J. Mao , K. K. McKee , P. D. Yurchenco , A. M. Mercurio , Genes Dev. 2015, 29, 1. 25561492 10.1101/gad.253682.114 PMC4281560

[37] X. Tang , Y. Hou , G. Yang , X. Wang , S. Tang , Y. E. Du , L. Yang , T. Yu , H. Zhang , M. Zhou , S. Wen , L. Xu , M. Liu , Cell Death Differ. 2016, 23, 132. 26068592 10.1038/cdd.2015.78 PMC4815985

[38] L. S. Steelman , T. Fitzgerald , K. Lertpiriyapong , L. Cocco , M. Y. Follo , A. M. Martelli , L. M. Neri , S. Marmiroli , M. Libra , S. Candido , F. Nicoletti , A. Scalisi , C. Fenga , L. Drobot , D. Rakus , A. Gizak , P. Laidler , J. Dulinska‐Litewka , J. Basecke , S. Mijatovic , D. Maksimovic‐Ivanic , G. Montalto , M. Cervello , M. Milella , A. Tafuri , Z. Demidenko , S. L. Abrams , J. A. McCubrey , Curr. Pharm. Des. 2016, 22, 2358. 26947958 10.2174/1381612822666160304151011

[39] R. Gargini , J. P. Cerliani , M. Escoll , I. M. Anton , F. Wandosell , Stem Cells 2015, 33, 646. 25407338 10.1002/stem.1904

[40] Y. Sun , A. C. Hedman , X. Tan , N. J. Schill , R. A. Anderson , Dev. Cell 2013, 25, 144. 23602387 10.1016/j.devcel.2013.03.010 PMC3740164

[41] A. Tomas , C. E. Futter , E. R. Eden , Trends Cell Biol. 2014, 24, 26. 24295852 10.1016/j.tcb.2013.11.002 PMC3884125

[42] A. Conte , S. Sigismund , Prog. Mol. Biol. Transl. Sci. 2016, 141, 225. 27378759 10.1016/bs.pmbts.2016.03.002

[43] L. Menard , N. Floc'h , M. J. Martin , D. A. E. Cross , Cancer Res. 2018, 78, 3267. 29555874 10.1158/0008-5472.CAN-17-2195

[44] L. von Kleist , W. Stahlschmidt , H. Bulut , K. Gromova , D. Puchkov , M. J. Robertson , K. A. MacGregor , N. Tomilin , A. Pechstein , N. Chau , M. Chircop , J. Sakoff , J. P. von Kries , W. Saenger , H. G. Krausslich , O. Shupliakov , P. J. Robinson , A. McCluskey , V. Haucke , Cell 2011, 146, 471. 21816279 10.1016/j.cell.2011.06.025

[45] A. Sorkin , L. K. Goh , Exp. Cell Res. 2009, 315, 683.1927803010.1016/j.yexcr.2008.07.029

[46] C. B. Thien , W. Y. Langdon , Biochem. J. 2005, 391, 153. 16212556 10.1042/BJ20050892 PMC1276912

[47] X. Jiang , F. Huang , A. Marusyk , A. Sorkin , Mol. Biol. Cell 2003, 14, 858. 12631709 10.1091/mbc.E02-08-0532 PMC151565

[48] H. Waterman , M. Katz , C. Rubin , K. Shtiegman , S. Lavi , A. Elson , T. Jovin , Y. Yarden , EMBO J. 2002, 21, 303. 11823423 10.1093/emboj/21.3.303 PMC125825

[49] L. M. Grovdal , E. Stang , A. Sorkin , I. H. Madshus , Exp. Cell Res. 2004, 300, 388. 15475003 10.1016/j.yexcr.2004.07.003

[50] G. Levkowitz , H. Waterman , E. Zamir , Z. Kam , S. Oved , W. Y. Langdon , L. Beguinot , B. Geiger , Y. Yarden , Genes Dev. 1998, 12, 3663. 9851973 10.1101/gad.12.23.3663 PMC317257

[51] H. Waterman , G. Levkowitz , I. Alroy , Y. Yarden , J. Biol. Chem. 1999, 274, 22151. 10428778 10.1074/jbc.274.32.22151

[52] T. G. Bivona , H. Hieronymus , J. Parker , K. Chang , M. Taron , R. Rosell , P. Moonsamy , K. Dahlman , V. A. Miller , C. Costa , G. Hannon , C. L. Sawyers , Nature 2011, 471, 523. 21430781 10.1038/nature09870 PMC3541675

[53] M. L. Sos , M. Koker , B. A. Weir , S. Heynck , R. Rabinovsky , T. Zander , J. M. Seeger , J. Weiss , F. Fischer , P. Frommolt , K. Michel , M. Peifer , C. Mermel , L. Girard , M. Peyton , A. F. Gazdar , J. D. Minna , L. A. Garraway , H. Kashkar , W. Pao , M. Meyerson , R. K. Thomas , Cancer Res. 2009, 69, 3256. 19351834 10.1158/0008-5472.CAN-08-4055 PMC2849653

[54] J. Rotow , T. G. Bivona , Nat. Rev. Cancer 2017, 17, 637. 29068003 10.1038/nrc.2017.84

[55] G. von Minckwitz , W. Jonat , P. Fasching , A. du Bois , U. Kleeberg , H. J. Luck , E. Kettner , J. Hilfrich , W. Eiermann , J. Torode , A. Schneeweiss , Breast Cancer Res. Treat. 2005, 89, 165. 15692759 10.1007/s10549-004-1720-2

[56] R. Dent , M. Trudeau , K. I. Pritchard , W. M. Hanna , H. K. Kahn , C. A. Sawka , L. A. Lickley , E. Rawlinson , P. Sun , S. A. Narod , Clin. Cancer Res. 2007, 13, 4429. 17671126 10.1158/1078-0432.CCR-06-3045

[57] G. Viale , N. Rotmensz , P. Maisonneuve , L. Bottiglieri , E. Montagna , A. Luini , P. Veronesi , M. Intra , R. Torrisi , A. Cardillo , E. Campagnoli , A. Goldhirsch , M. Colleoni , Breast Cancer Res. Treat. 2009, 116, 317. 18839307 10.1007/s10549-008-0206-z

[58] H. Zhang , B. Han , H. Lu , Y. Zhao , X. Chen , Q. Meng , M. Cao , L. Cai , J. Hu , Cancer Lett. 2018, 433, 186. 29981430 10.1016/j.canlet.2018.07.002

[59] X. L. Yan , C. J. Fu , L. Chen , J. H. Qin , Q. Zeng , H. F. Yuan , X. Nan , H. X. Chen , J. N. Zhou , Y. L. Lin , X. M. Zhang , C. Z. Yu , W. Yue , X. T. Pei , Breast Cancer Res. Treat. 2012, 132, 153. 21584665 10.1007/s10549-011-1577-0

[60] S. K. Yeo , J. Wen , S. Chen , J. L. Guan , Cancer Res. 2016, 76, 3397. 27197172 10.1158/0008-5472.CAN-15-2946 PMC4990205

[61] P. Savage , A. Blanchet‐Cohen , T. Revil , D. Badescu , S. M. I. Saleh , Y. C. Wang , D. Zuo , L. Liu , N. R. Bertos , V. Munoz‐Ramos , M. Basik , K. Petrecca , J. Asselah , S. Meterissian , M. C. Guiot , A. Omeroglu , C. L. Kleinman , M. Park , J. Ragoussis , Cell Rep. 2017, 21, 1140. 29091754 10.1016/j.celrep.2017.10.015

[62] Z. Zheng , N. Shao , H. Weng , W. Li , J. Zhang , L. Zhang , L. Yang , S. Ye , Med. Oncol. 2015, 32, 275. 25429827 10.1007/s12032-014-0275-2 PMC4246130

[63] R. Wise , A. Zolkiewska , Breast Cancer Res. Treat. 2017, 166, 421. 28791489 10.1007/s10549-017-4440-0 PMC5669811

[64] J. M. Balko , L. J. Schwarz , N. E. Bhola , R. Kurupi , P. Owens , T. W. Miller , H. Gomez , R. S. Cook , C. L. Arteaga , Cancer Res. 2013, 73, 6346. 23966295 10.1158/0008-5472.CAN-13-1385 PMC4090144

[65] M. Luo , L. Shang , M. D. Brooks , E. Jiagge , Y. Zhu , J. M. Buschhaus , S. Conley , M. A. Fath , A. Davis , E. Gheordunescu , Y. Wang , R. Harouaka , A. Lozier , D. Triner , S. McDermott , S. D. Merajver , G. D. Luker , D. R. Spitz , M. S. Wicha , Cell Metab. 2018, 28, 69. 29972798 10.1016/j.cmet.2018.06.006 PMC6037414

[66] T. Wang , J. F. Fahrmann , H. Lee , Y. J. Li , S. C. Tripathi , C. Yue , C. Zhang , V. Lifshitz , J. Song , Y. Yuan , G. Somlo , R. Jandial , D. Ann , S. Hanash , R. Jove , H. Yu , Cell Metab. 2018, 27, 136. 29249690 10.1016/j.cmet.2017.11.001 PMC5777338

[67] S. Ye , Y. F. Ding , W. H. Jia , X. L. Liu , J. Y. Feng , Q. Zhu , S. L. Cai , Y. S. Yang , Q. Y. Lu , X. T. Huang , J. S. Yang , S. N. Jia , G. P. Ding , Y. H. Wang , J. J. Zhou , Y. D. Chen , W. J. Yang , Cancer Res. 2019, 79, 4729. 31308046 10.1158/0008-5472.CAN-19-1084

[68] F. Zheng , C. Yue , G. Li , B. He , W. Cheng , X. Wang , M. Yan , Z. Long , W. Qiu , Z. Yuan , J. Xu , B. Liu , Q. Shi , E. W. Lam , M. C. Hung , Q. Liu , Nat. Commun. 2016, 7, 10180. 26782714 10.1038/ncomms10180 PMC4735655

[69] I. Eke , N. Cordes , Semin. Cancer Biol. 2015, 31, 65. 25117005 10.1016/j.semcancer.2014.07.009

[70] A. W. Holle , J. L. Young , J. P. Spatz , Adv. Drug Delivery Rev. 2016, 97, 270. 10.1016/j.addr.2015.10.007 26485156

[71] G. Oktem , O. Sercan , U. Guven , R. Uslu , A. Uysal , G. Goksel , S. Ayla , A. Bilir , Oncol. Rep. 2014, 32, 641. 24927163 10.3892/or.2014.3252

[72] R. Januchowski , M. Swierczewska , K. Sterzynska , K. Wojtowicz , M. Nowicki , M. Zabel , J. Cancer 2016, 7, 1295. 27390605 10.7150/jca.15371 PMC4934038

[73] M. Najafi , A. Ahmadi , K. Mortezaee , Cell Biol. Int. 2019, 43, 1206. 31136035 10.1002/cbin.11187

[74] E. Barbieri , P. P. Di Fiore , S. Sigismund , Curr. Opin. Cell Biol. 2016, 39, 21. 26872272 10.1016/j.ceb.2016.01.012

[75] C. H. Lee , Y. T. Wu , H. C. Hsieh , Y. Yu , A. L. Yu , W. W. Chang , Biochimie 2014, 104, 117. 24950183 10.1016/j.biochi.2014.06.011

[76] S. Sigismund , V. Algisi , G. Nappo , A. Conte , R. Pascolutti , A. Cuomo , T. Bonaldi , E. Argenzio , L. G. Verhoef , E. Maspero , F. Bianchi , F. Capuani , A. Ciliberto , S. Polo , P. P. Di Fiore , EMBO J. 2013, 32, 2140. 23799367 10.1038/emboj.2013.149 PMC3730230

[77] S. Sigismund , E. Argenzio , D. Tosoni , E. Cavallaro , S. Polo , P. P. Di Fiore , Dev. Cell 2008, 15, 209. 18694561 10.1016/j.devcel.2008.06.012

[78] BIG Data Center Members , Nucleic Acids Res. 2018, 46, D14. 29036542 10.1093/nar/gkx897 PMC5753194

[79] J. Cao , M. Spielmann , X. Qiu , X. Huang , D. M. Ibrahim , A. J. Hill , F. Zhang , S. Mundlos , L. Christiansen , F. J. Steemers , C. Trapnell , J. Shendure , Nature 2019, 566, 496. 30787437 10.1038/s41586-019-0969-x PMC6434952

[80] A. T. Lun , D. J. McCarthy , J. C. Marioni , F1000Research 2016, 5, 2122. 27909575 10.12688/f1000research.9501.1 PMC5112579

[81] K. Shien , S. Toyooka , H. Yamamoto , J. Soh , M. Jida , K. L. Thu , S. Hashida , Y. Maki , E. Ichihara , H. Asano , K. Tsukuda , N. Takigawa , K. Kiura , A. F. Gazdar , W. L. Lam , S. Miyoshi , Cancer Res. 2013, 73, 3051. 23542356 10.1158/0008-5472.CAN-12-4136 PMC4506773

