# Supplementary material for: SGCE Promotes Breast Cancer Stem Cells by Stabilizing EGFR
Source: Adv Sci (Weinh). 2020 Jun 8;7(14):1903700. doi: 10.1002/advs.201903700 (PMC7375232; doi:10.1002/advs.201903700)
Supplement: Supplementary file 1 — Supporting Information [file ADVS-7-1903700-s001.pdf]

Table S1. Correlations among SGCE and differentially expressed genes (DEGs) in $CD24^{low}CD44^{high}$ $ALDH1^{+}$ BCSCs.

Gene name	Pearson-correlation	*P* value	Sample size
C1R	0.415	9.59E-47	1 093
KIT	0.4116	6.16E-46	1 093
FZD7	0.3755	6.30E-38	1 093
NTRK3	0.3614	4.72E-35	1 093
LAMA2	0.3503	6.53E-33	1 093
LRP1	0.3501	7.11E-33	1 093
EGFR	0.345	6.47E-32	1 093
BMP2	0.3356	3.53E-30	1 093
PTCH1	0.3287	5.85E-29	1 093
SHC4	0.3282	7.38E-29	1 093
COL5A1	0.3251	2.53E-28	1 093
COL1A1	0.3218	9.44E-28	1 093
WNT5B	0.3218	9.29E-28	1 093
TUBB2B	0.3018	1.91E-24	1 093
IL1R1	0.2837	1.13E-21	1 093
EFNA5	0.2678	2.09E-19	1 093
ELF5	0.2651	4.92E-19	1 093
DAPK1	0.2528	2.15E-17	1 093
IL6	0.2515	3.13E-17	1 093
FZD9	0.2372	1.92E-15	1 093
TLR2	0.233	6.06E-15	1 093
SERPINE1	0.233	6.18E-15	1 093
PLCE1	0.2281	2.29E-14	1 093
SOCS3	0.2206	1.61E-13	1 093
THBS1	0.2159	5.46E-13	1 093

Genes are highly expressed in $CD24^{low}CD44^{high}ALDH1^{+}$ BCSCs [1]. We analyzed correlations among SGCE and target genes using LinkedOmics (http://www.linkedomics.org/login.php) [2].

Table S2. Primers and shRNA sequences used in this paper.

Primer	Primer sequence
SGCE-sh#1-F	CCGGACCTGGATGGCTTCGATATATCTCGAGATATA TCGAAGCCATCCAGGTTTTTG
SGCE-sh#1-R	AATTCAAAAAACCTGGATGGCTTCGATATATCTCG AGATATATCGAAGCCATCCAGGT
SGCE-sh#2-F	CCGGATTGAGATGTAGTCAAGAAATCTCGAGATTT CTTGACTACATCTCAA TTTTTTG

SGCE-sh#2-R	AATTCAAAAAATTGAGATGTAGTCAAGAAATCTCG AGA TTTCTTGACTACATCTCAAT
sh-luc-F	GATCCGTCCAGATTGTCCGCAACTACTTCCTGTCA GATAGTTGCGGACAATCTGGACTTTTTG
sh-luc-R	AATTCAAAAAGTCCAGATTGTCCGCAACTATCTGA CAGGAAGTAGTTGCGGACAATCTGGACG
sh-c-Cbl-F	GATCCGAGCTTTCGACAGGCTCTACTTCCTGTCAG ATAGAGCCTGTCGAAAGCTCTTTTTG
sh-c-Cbl-R	AATTCAAAAAGAGCTTTCGACAGGCTCTA TCTGACAGGAAGTAGAGCCTGTCGAAAGCTCG
EGFR-sh#1-F	GATCCGAACATAACATCCTTGGGATTCTTCCTGTC AGAAATCCCAAGGATGTTATGTTCTTTTTG
EGFR-sh#1-R	AATTCAAAAAGAACATAACATCCTTGGGATTTCTG ACAGGAAGAATCCCAAGGATGTTATGTTCG
EGFR-sh#2-F	GATCCGAAGGAAACTGAATTCAAATTCTTCCTGTC AGAAATTTGAATTCAGTTTCCTTCTTTTTG-
EGFR-sh#2-R	AATTCAAAAAGAAGGAAACTGAATTCAAATTTCT GACAGGAAGAATTTGAATTCAGTTTCCTTCG
siSGCE#1-F	GGAAAUCAUACCUCCUUUATT
siSGCE#1-R	TTCCUUUAGUAUGGAGGAAAU
siSGCE#2-F	ACCUGGAUGGCUUCGAUAUAUTT
siSGCE#2-R	TTUGGACCUACCGAAGCUAUAUA
q-RT-SGCE-F	GGTTCTTGGAGACTTTCTTGGC
q-RT-SGCE-R	ATGACATAAACGCCCTCCTTC
q-RT-ITGA3-F	CTACCACAACGAGATGTGCAA
q-RT-ITGA3-R	CCGAAGTACACAGTGTTCTGG
q-RT-ITGA6-F	GGCGGTGTTATGTCCTGAGTC
q-RT-ITGA6-R	AATCGCCCATCACAAAAGCTC
q-RT-ITGB4-F	CTCCACCGAGTCAGCCTTC

q-RT-ITGB4-R	CGGGTAGTCCTGTGTCCTGTA
q-RT-LAMA3-F	CACCGGGATATTTCGGGAATC
q-RT-LAMA3-R	AGCTGTCGCAATCATCACATT
q-RT-LAMB3-F	CCAAAGGTGCGACTGCAATG
q-RT-LAMB3-R	AGTTCTTGCCTTCGGTGTGG
q-RT-LAMC2-F	CAAAGGTTCTCTTAGTGCTCGAT
q-RT-LAMC2-R	CACTTGGAGTCTAGCAGTCTCT
q-RT-FN-F	GAGAATGGACCTGCAAGCCCA
q-RT-FN-R	AGTGCAAGTGATGCGTCCGC
q-RT-COL17A1-F	ACCAGCAATGGCTATGCTAAAA
q-R -COL17A1-R	GCCTCGTGTGCTTCCAGTT
q-RT-GAPDH-F	AGCCACATCGCTCAGACAC
q-RT-GAPDH-R	GCCCAATACGACCAAATCC
q-RT-ACTA2-F:	CTATGAGGGCTATGCCTTGCC
q-RT-ACTA2-R	GCTCAGCAGTAGTAACGAAGGA
q-RT-S100A2-F:	GCCAAGAGGGCGACAAGTT
q-RT-S100A2-R	AGGAAAACAGCATACTCCTGGA
q-RT-S100A4-F:	GATGAGCAACTTGGACAGCAA
q-RT-S100A4-R	CTGGGCTGCTTATCTGGGAAG

[1] M. Liu, Y. Liu, L. Deng, D. Wang, X. He, L. Zhou, M. S. Wicha, F. Bai, S. Liu, *Mol. Cancer* **2018**, 17, 65.

[2] S. V. Vasaikar, P. Straub, J. Wang, B. Zhang, *Nucleic Acids Res* **2018**, 46, D956.

Figure S1. UMAP plots of TNBC epithelial cells.

A-G) UMAP plots of TNBC epithelial cells showing expression levels of CD24, CD44, ALDH1A3, SGCE, EGFR, ACVR2A, and PLXDC2.

Figure S2. SGCE promotes self-renewal of BCSCs.

A-B) Relapse-free survival (RFS) (A) and distal metastasis-free survival (DMFS) (B) of SGCE in various types of breast cancer. C) Relative expression of SGCE in various types of breast cancer using TCGA database. D) Assay of $CD24^{low}CD44^{high}$ population upon SGCE knockdown in HCC1937 cells. E) Knockdown efficiencies of SGCE in TNBC cell lines. F) Overexpression efficiencies of SGCE by PCDH plasmid containing Flag tag in TNBC cell lines. (**) $P < 0.01$.

Figure S3. Loss of SGCE increases chemotherapy sensitivity of breast cancer through BCSCs.

A-B) Assay of $ALDH^{+}$ population upon SGCE knockdown in HCC1806 (A) and MDA-MB-231 cells (B). C) Assay of $CD24^{low}CD44^{high}$ population upon SGCE knockdown in HCC1806 cells. D-E) Clonal formation assay upon SGCE knockdown in HCC1806 (D) and MDA-MB-231 (E) cells. F-G) Tumorsphere assay upon SGCE knockdown in HCC1806 (F) and MDA-MB-231 (G) cells. H-I) Cell viability (%) in HCC1806 (H) and MDA-MB-231 cells (I) after treatment with various concentrations of cisplatin. J-Q) Tumorsphere (J), clonal formation (K), ALDH ratio (L), $CD24^{low}CD44^{high}$ ratio (M), and their percentage calculations (N for J, O for K, P for L, and Q for M) following treatment with doxorubicin, paclitaxel, and cisplatin in SGCE-depleted HCC1806 cells. (*) $P < 0.05$; (**) $P < 0.01$. Dox: doxorubicin; Ptx: paclitaxel; Cis: cisplatin.

Figure S4. SGCE promotes ECM deposition.

A) Co-expression network of SGCE and ECM-related genes in basal-like breast cancer based on TCGA database. B) Heatmap of ECM receptor interaction in SGCE-knockdown HCC1806 cells. C) KEGG pathway analysis of SGCE in basal-like patients from TCGA database. Top 10% positively related genes with SGCE were selected for KEGG pathway analysis. D) KEGG pathway analysis in SGCE-knockdown HCC1806 cells.

Figure S5. SGCE regulates BCSCs and ECM through EGFR.

A) Western blot of EGFR and related genes in SGCE-knockdown MDA-MB-231 cells. B-D) ALDH (B), tumorsphere (C), and clonal formation (D) analyses with EGFR overexpression in SGCE-knockdown MDA-MB-231 cells. E) mRNA levels of ECM-related genes in EGFR knockdown cells. F) Protein levels in ECM-related genes in SGCE-depleted cells with EGFR overexpression in MDA-MB-231 cells. (*) $P < 0.05$; (**) $P < 0.01$; (***) $P < 0.001$.

Figure S6. SGCE regulates EGFR lysosomal degradation.

A) Immunoblotting analysis of EGFR in SGCE-knockdown cells with internalization inhibitors. B) Immunoblotting analysis of EGFR in SGCE-knockdown MDA-MB-231 cells with NH_4Cl treatment. C) FACS analysis of EGFR in SGCE-knockdown MDA-MB-231 cells with NH_4Cl treatment. D) Immunofluorescence staining of EGFR and LAMP1 in MDA-MB-231 cells. E-F) Immunoblotting analysis of EGFR in SGCE-knockdown MDA-MB-231 (E) and HCC1806 (F) cells after MG132

treatment. G) Negative co-immunoprecipitation results of EGFR and SGCE. H) Negative co-immunoprecipitation results of EGFR and GRB2.

Figure S7. SGCE promotes drug resistance-targeted EGFR.

A) GSEA of NF-kB signaling pathway in SGCE-knockdown HCC1806 cell lines. B-C) Immunoblotting analysis of c-Met in SGCE-knockdown cell lines. D-E) Immunoblotting analysis of NF-kB signaling pathway in SGCE-knockdown TNBC cell lines. F-I) Drug sensitivity experiments in gefitinib- and lapatinib-resistant cells in comparison to parental cells. J-K) Drug sensitivity experiments following SGCE knockdown in lapatinib-resistant HCC1806 (J) and MDA-MB-231 (K) cell lines.

Figure S8. Signaling pathways regulated by SGCE.

A) Luciferase activities with overexpression of SGCE. HEK293 cells were co-transfected with SGCE, luciferase-responsive plasmid, and Renilla (10:10:1, total 0.22 μg). Cells were collected with dual-luciferase reporter assay system. B-C) Immunoblotting analysis of stemness-related genes in SGCE-knockdown cell lines. D-E) NF-kB signaling pathway in TNBC cell lines. F) Immunoblotting analysis of EGFR in gefitinib-resistant cells. G) Co-immunoprecipitation results of EGFR and c-Cbl in gefitinib-resistant cells.

Fig. S1

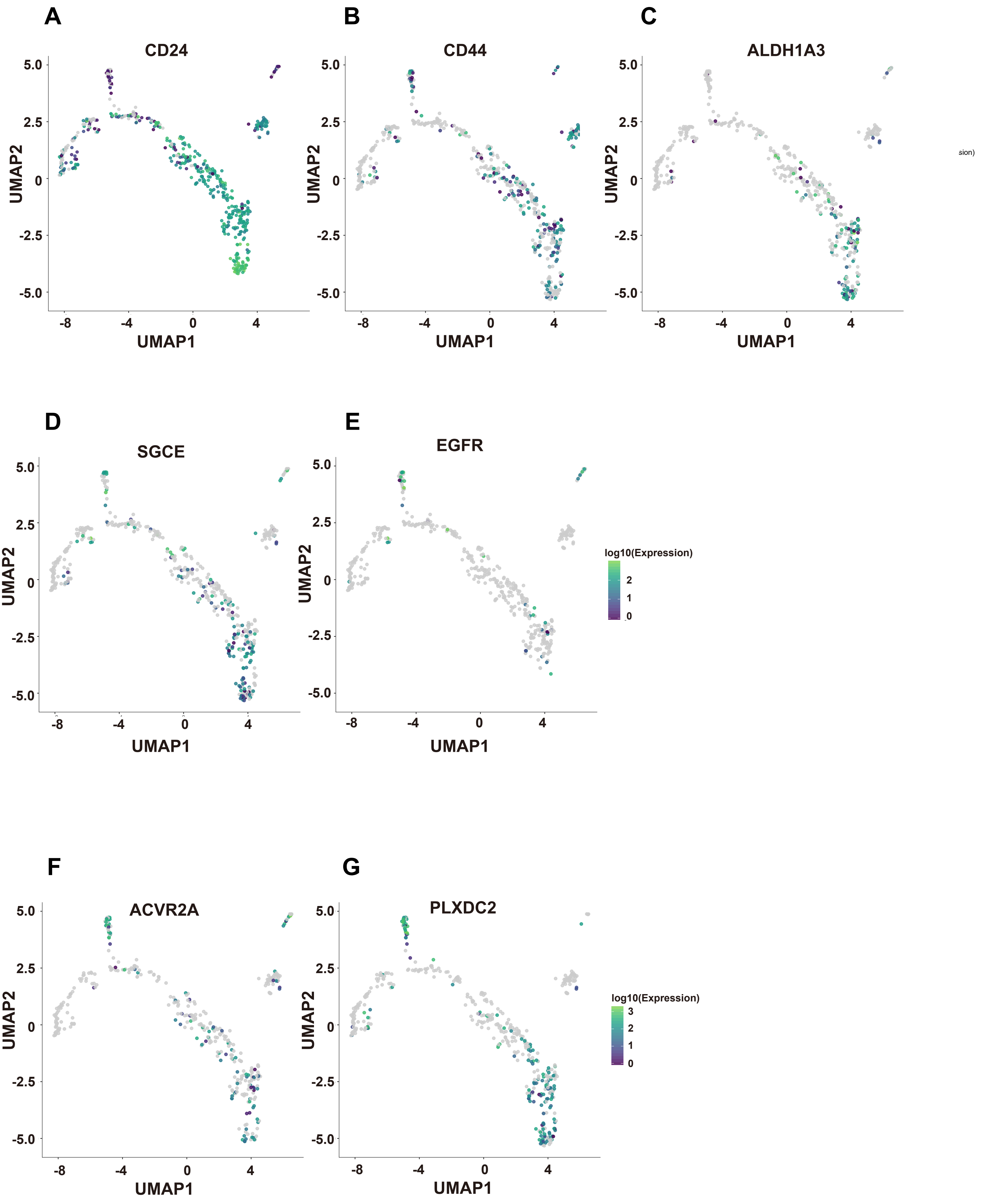
A
CD24
B
CD44
C
ALDH1A3
D
SGCE
E
EGFR
F
ACVR2A
G
PLXDC2
UMAP1
UMAP2
log10(Expression)

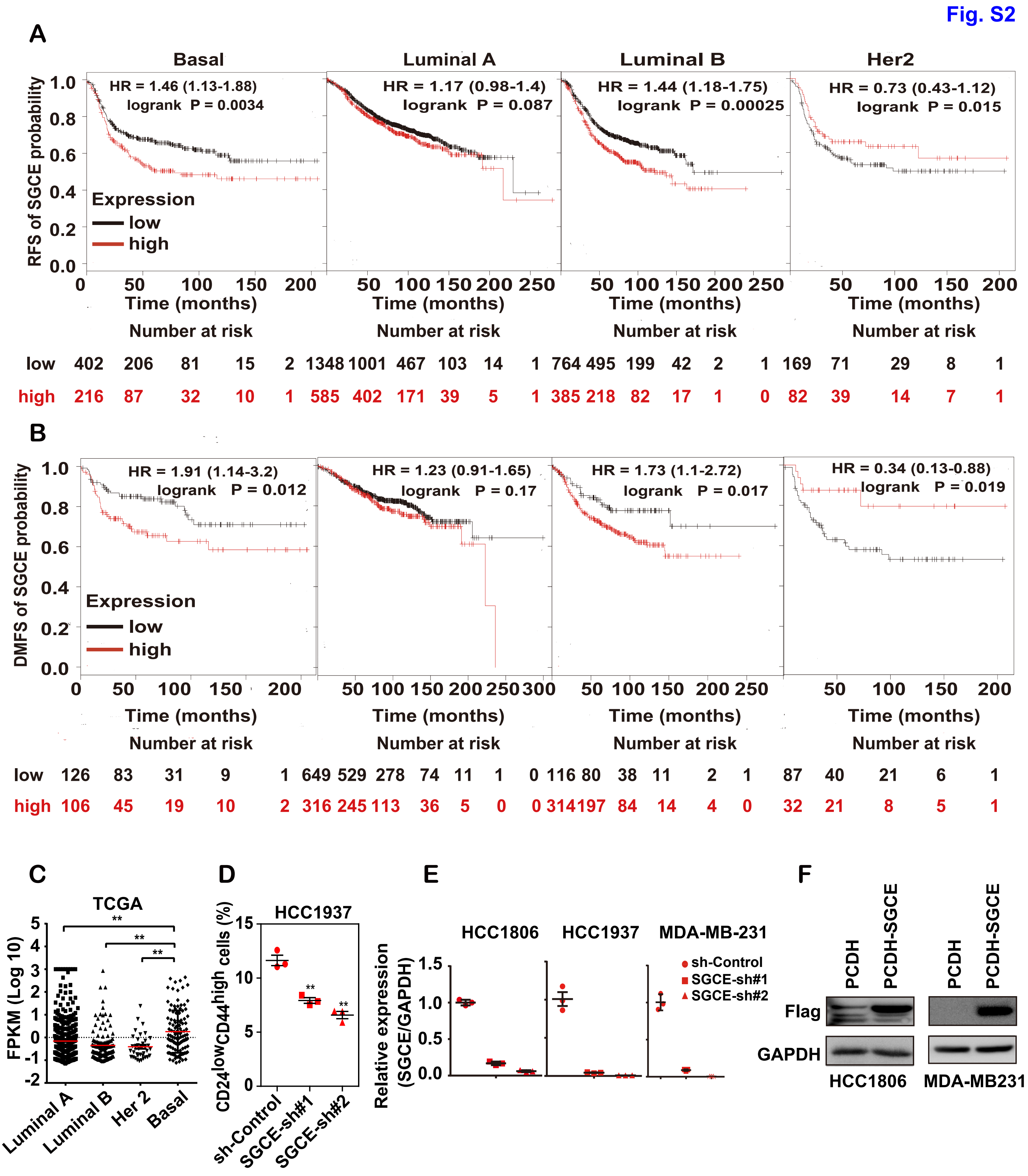
Fig. S2
A
Basal
Luminal A
Luminal B
Her2
HR = 1.46 (1.13-1.88) logrank P = 0.0034
HR = 1.17 (0.98-1.4) logrank P = 0.087
HR = 1.44 (1.18-1.75) logrank P = 0.00025
HR = 0.73 (0.43-1.12) logrank P = 0.015
RFS of SGCE probability
Expression
low
high
Time (months)
Number at risk
low 402 206 81 15 2 1348 1001 467 103 14 1 764 495 199 42 2 1 169 71 29 8 1
high 216 87 32 10 1 585 402 171 39 5 1 385 218 82 17 1 0 82 39 14 7 1
B
HR = 1.91 (1.14-3.2) logrank P = 0.012
HR = 1.23 (0.91-1.65) logrank P = 0.17
HR = 1.73 (1.1-2.72) logrank P = 0.017
HR = 0.34 (0.13-0.88) logrank P = 0.019
DMFS of SGCE probability
Expression
low
high
Time (months)
Number at risk
low 126 83 31 9 1 649 529 278 74 11 1 0 116 80 38 11 2 1 87 40 21 6 1
high 106 45 19 10 2 316 245 113 36 5 0 0 314 197 84 14 4 0 32 21 8 5 1
C
TCGA
FPKM (Log 10)
Luminal A
Luminal B
Her 2
Basal
D
HCC1937
CD24lowCD44high cells (%)
sh-Control
SGCE-sh#1
SGCE-sh#2
E
HCC1806
HCC1937
MDA-MB-231
Relative expression (SGCE/GAPDH)
sh-Control
SGCE-sh#1
SGCE-sh#2
F
PCDH
PCDH-SGCE
Flag
GAPDH
HCC1806
MDA-MB231

Fig. S3

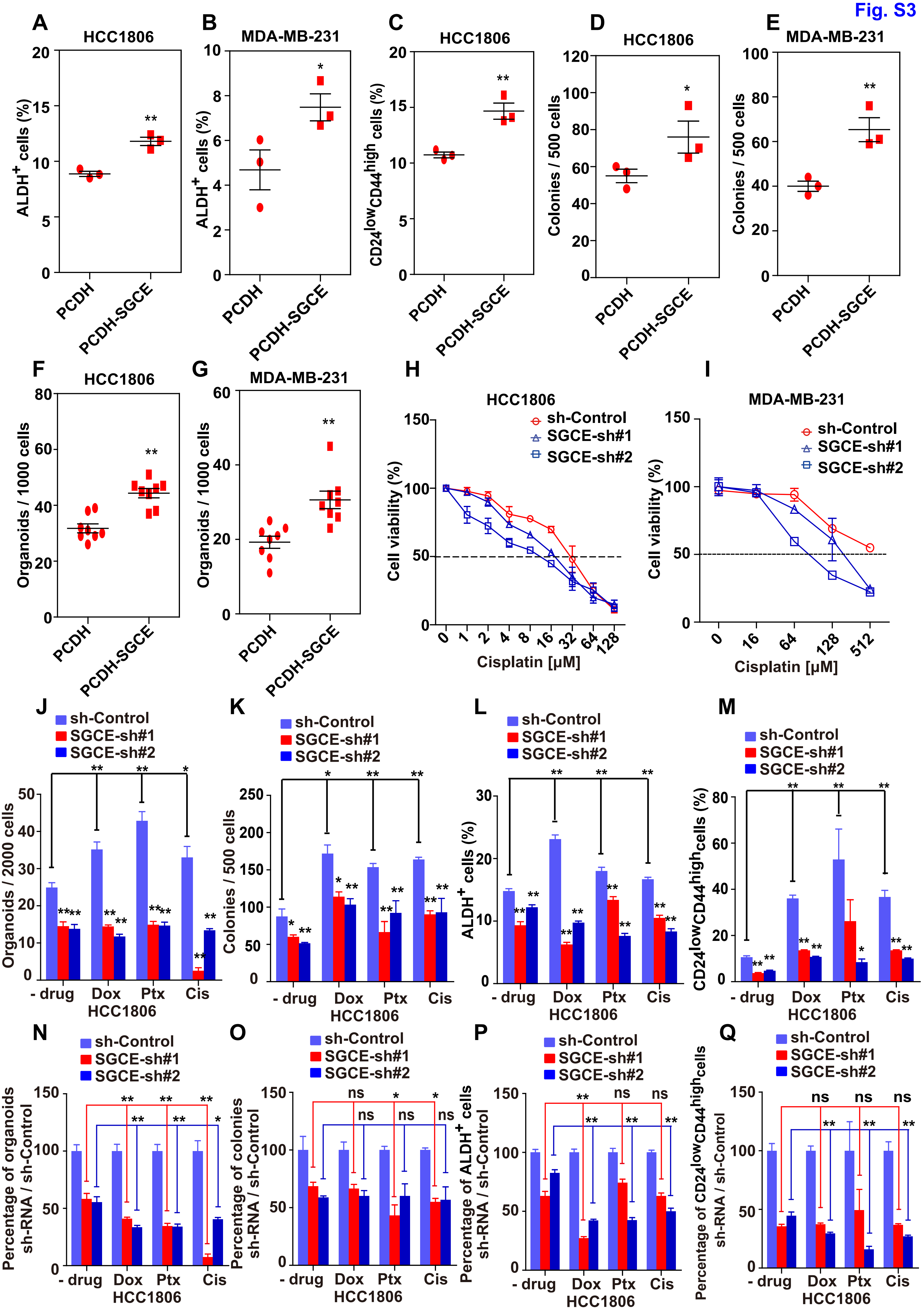

A
HCC1806
ALDH+ cells (%)
PCDH
PCDH-SGCE
B
MDA-MB-231
ALDH+ cells (%)
C
HCC1806
CD24lowCD44high cells (%)
D
HCC1806
Colonies / 500 cells
E
MDA-MB-231
Colonies / 500 cells
F
HCC1806
Organoids / 1000 cells
G
MDA-MB-231
Organoids / 1000 cells
H
HCC1806
sh-Control
SGCE-sh#1
SGCE-sh#2
Cell viability (%)
Cisplatin [μM]
I
MDA-MB-231
sh-Control
SGCE-sh#1
SGCE-sh#2
Cell viability (%)
Cisplatin [μM]
J
sh-Control
SGCE-sh#1
SGCE-sh#2
Organoids / 2000 cells
- drug
Dox
Ptx
Cis
HCC1806
K
Colonies / 500 cells
L
ALDH+ cells (%)
M
CD24lowCD44highcells (%)
N
Percentage of organoids sh-RNA / sh-Control
O
Percentage of colonies sh-RNA / sh-Control
P
Percentage of ALDH+ cells sh-RNA / sh-Control
Q
Percentage of CD24lowCD44highcells sh-RNA / sh-Control
ns

Fig. S4

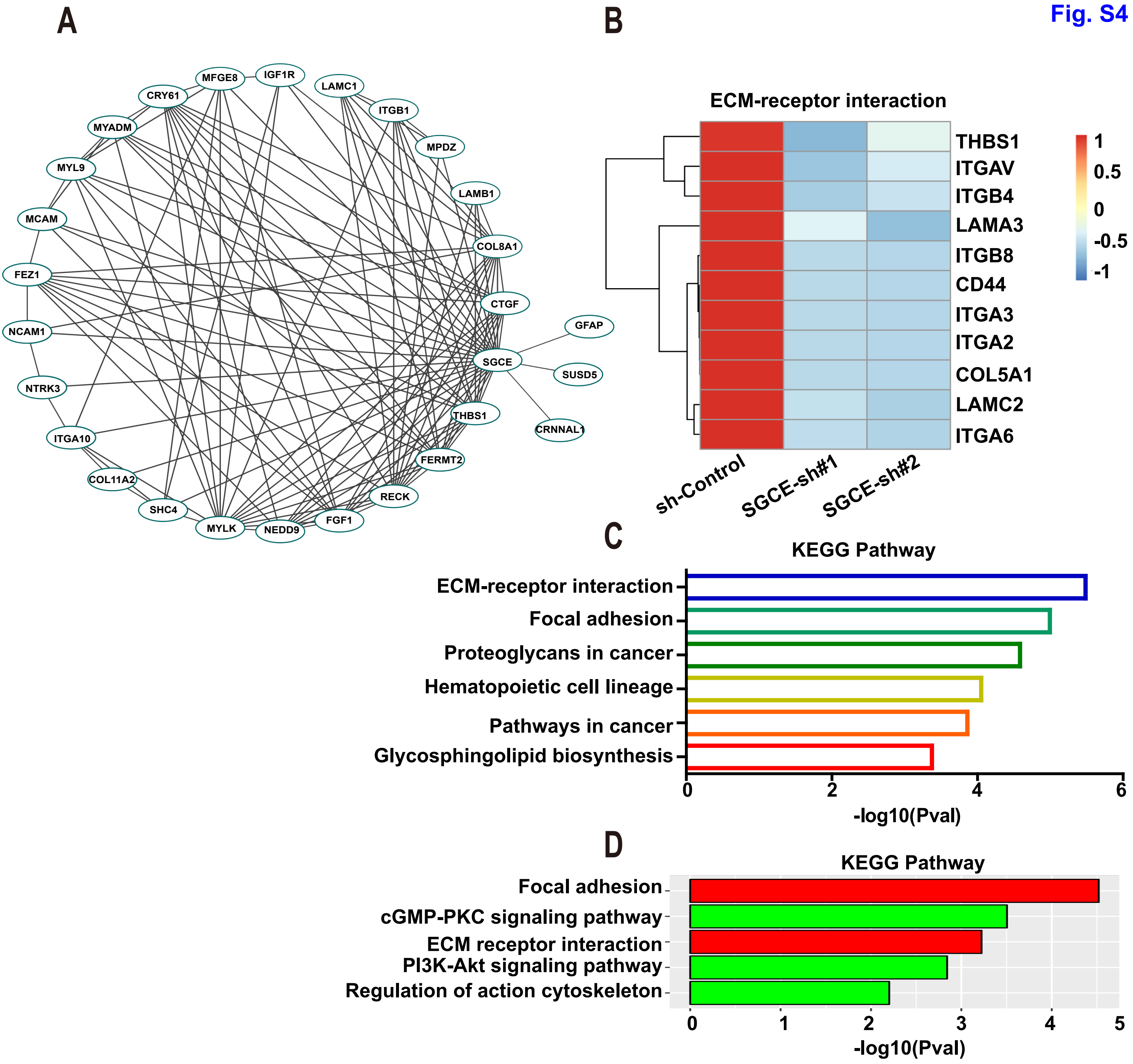

A
MFGE8
IGF1R
LAMC1
CRY61
ITGB1
MYADM
MPDZ
MYL9
LAMB1
MCAM
COL8A1
FEZ1
CTGF
GFAP
NCAM1
SGCE
SUSD5
NTRK3
THBS1
CRNNAL1
ITGA10
FERMT2
COL11A2
RECK
SHC4
MYLK
NEDD9
FGF1
B
ECM-receptor interaction
THBS1
ITGAV
ITGB4
LAMA3
ITGB8
CD44
ITGA3
ITGA2
COL5A1
LAMC2
ITGA6
sh-Control
SGCE-sh#1
SGCE-sh#2
1
0.5
0
-0.5
-1
C
KEGG Pathway
ECM-receptor interaction
Focal adhesion
Proteoglycans in cancer
Hematopoietic cell lineage
Pathways in cancer
Glycosphingolipid biosynthesis
0
2
4
6
-log10(Pval)
D
KEGG Pathway
Focal adhesion
cGMP-PKC signaling pathway
ECM receptor interaction
PI3K-Akt signaling pathway
Regulation of action cytoskeleton
0
1
2
3
4
5
-log10(Pval)

Fig. S5

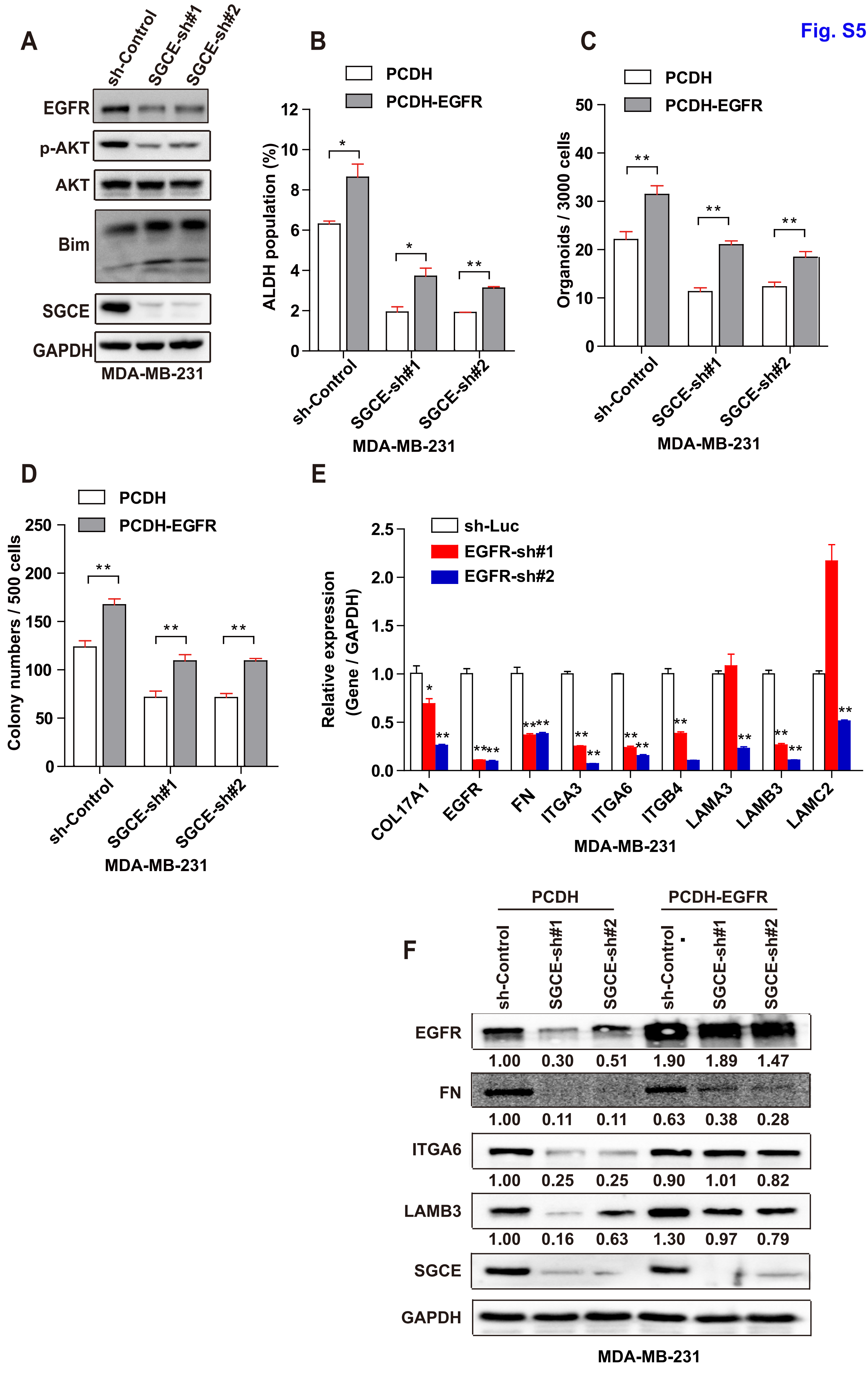

A
sh-Control
SGCE-sh#1
SGCE-sh#2
EGFR
p-AKT
AKT
Bim
SGCE
GAPDH
MDA-MB-231
B
PCDH
PCDH-EGFR
ALDH population (%)
sh-Control
SGCE-sh#1
SGCE-sh#2
MDA-MB-231
C
PCDH
PCDH-EGFR
Organoids / 3000 cells
sh-Control
SGCE-sh#1
SGCE-sh#2
MDA-MB-231
D
PCDH
PCDH-EGFR
Colony numbers / 500 cells
sh-Control
SGCE-sh#1
SGCE-sh#2
MDA-MB-231
E
sh-Luc
EGFR-sh#1
EGFR-sh#2
Relative expression (Gene / GAPDH)
COL17A1
EGFR
FN
ITGA3
ITGA6
ITGB4
LAMA3
LAMB3
LAMC2
MDA-MB-231
F
PCDH
PCDH-EGFR
sh-Control
SGCE-sh#1
SGCE-sh#2
sh-Control
SGCE-sh#1
SGCE-sh#2
EGFR
1.00 0.30 0.51 1.90 1.89 1.47
FN
1.00 0.11 0.11 0.63 0.38 0.28
ITGA6
1.00 0.25 0.25 0.90 1.01 0.82
LAMB3
1.00 0.16 0.63 1.30 0.97 0.79
SGCE
GAPDH
MDA-MB-231

Fig. S6

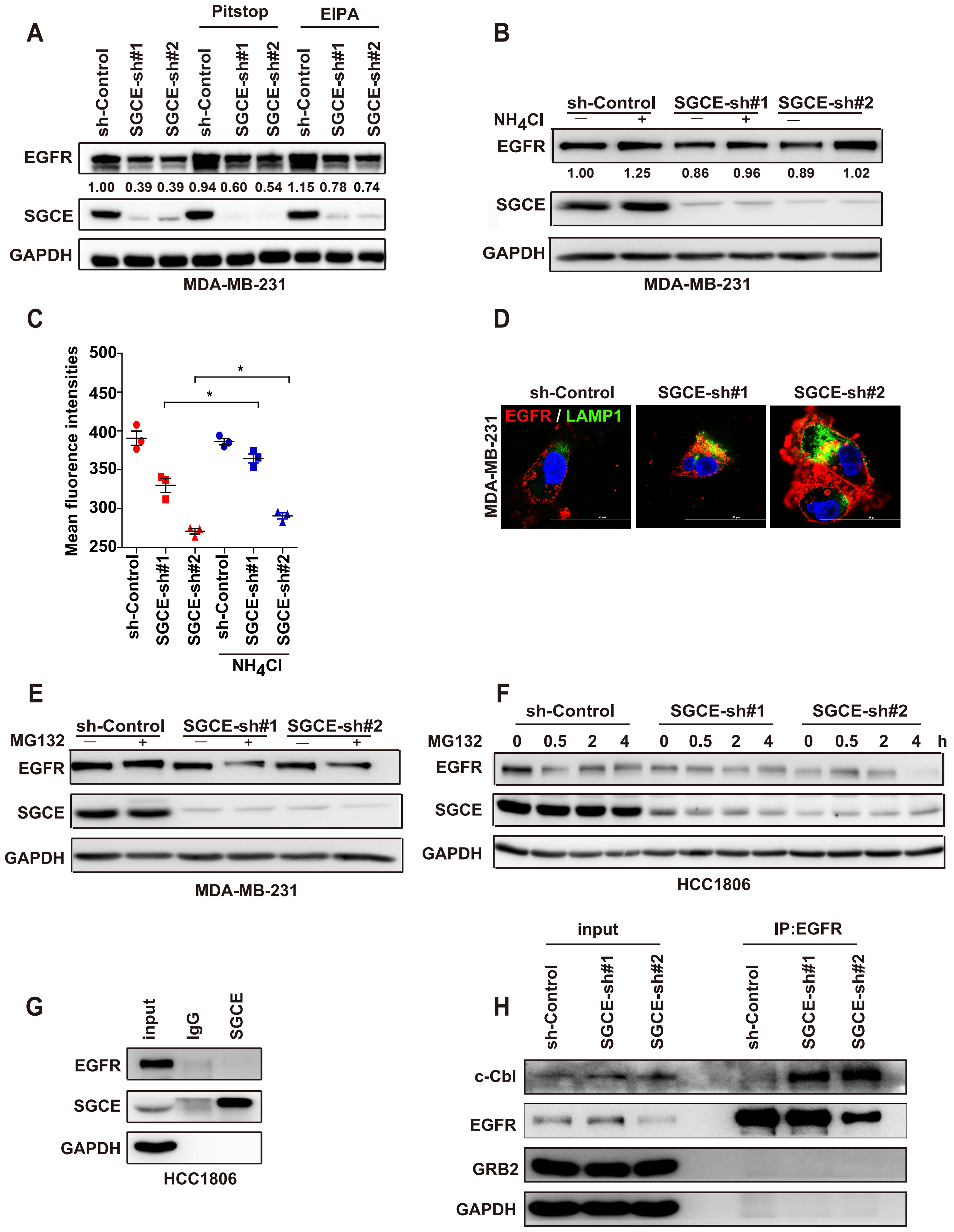

A
Pitstop
EIPA
sh-Control
SGCE-sh#1
SGCE-sh#2
EGFR
1.00 0.39 0.39 0.94 0.60 0.54 1.15 0.78 0.74
SGCE
GAPDH
MDA-MB-231
B
sh-Control
SGCE-sh#1
SGCE-sh#2
NH4Cl
EGFR
1.00 1.25 0.86 0.96 0.89 1.02
SGCE
GAPDH
MDA-MB-231
C
Mean fluorescence intensities
500
450
400
350
300
250
*
NH4Cl
D
sh-Control
SGCE-sh#1
SGCE-sh#2
EGFR / LAMP1
MDA-MB-231
E
MG132
sh-Control
SGCE-sh#1
SGCE-sh#2
EGFR
SGCE
GAPDH
MDA-MB-231
F
MG132
0 0.5 2 4 h
EGFR
SGCE
GAPDH
HCC1806
G
input
IgG
SGCE
EGFR
SGCE
GAPDH
HCC1806
H
input
IP:EGFR
c-Cbl
EGFR
GRB2
GAPDH

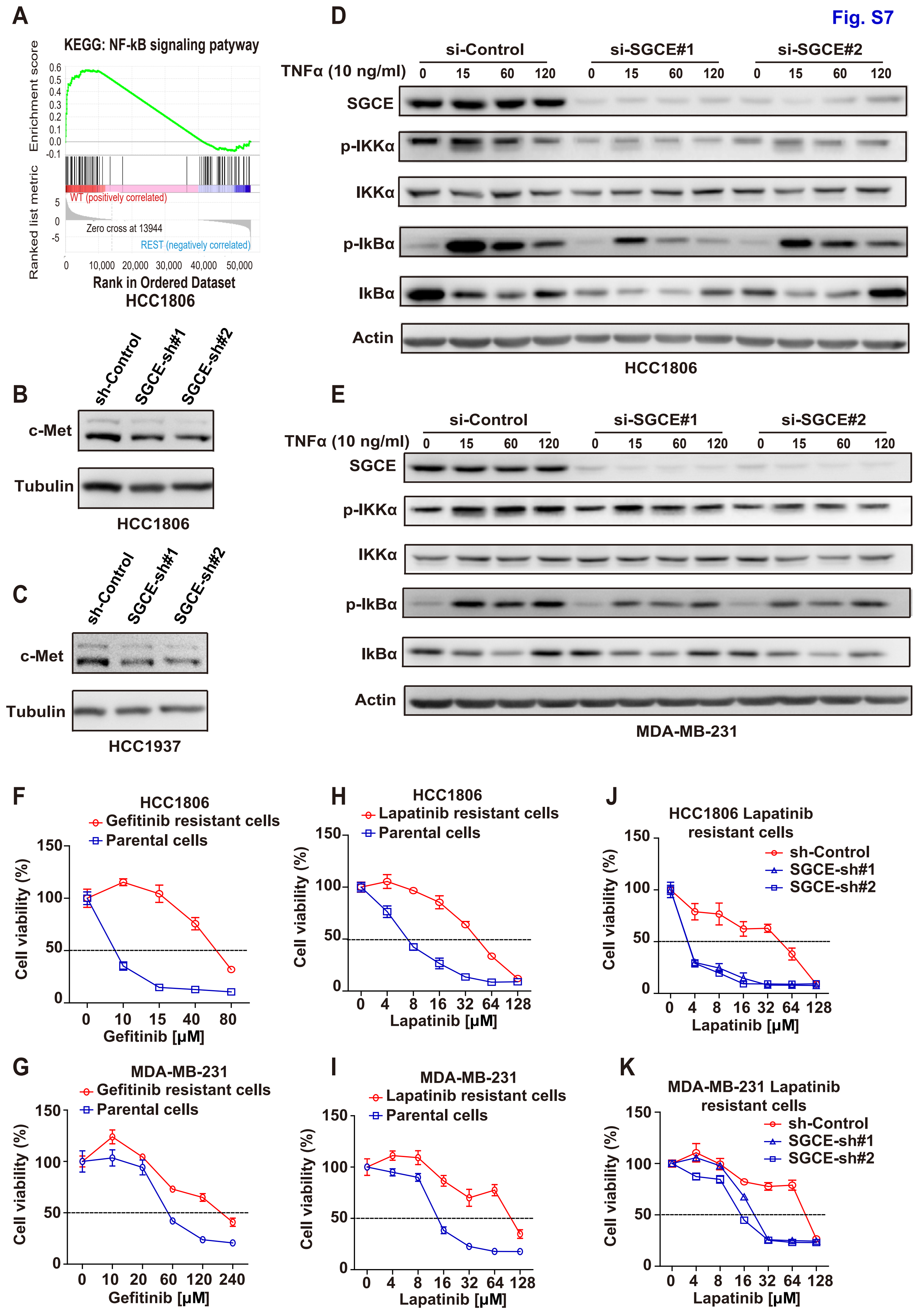
A
KEGG: NF-kB signaling patyway
Enrichment score
Ranked list metric
WT (positively correlated)
Zero cross at 13944
REST (negatively correlated)
Rank in Ordered Dataset
HCC1806
D
si-Control
si-SGCE#1
si-SGCE#2
TNFα (10 ng/ml) 0 15 60 120 0 15 60 120 0 15 60 120
SGCE
p-IKKα
IKKα
p-IkBα
IkBα
Actin
HCC1806
B
sh-Control
SGCE-sh#1
SGCE-sh#2
c-Met
Tubulin
HCC1806
E
si-Control
si-SGCE#1
si-SGCE#2
TNFα (10 ng/ml) 0 15 60 120 0 15 60 120 0 15 60 120
SGCE
p-IKKα
IKKα
p-IkBα
IkBα
Actin
MDA-MB-231
C
sh-Control
SGCE-sh#1
SGCE-sh#2
c-Met
Tubulin
HCC1937
F
HCC1806
Gefitinib resistant cells
Parental cells
Cell viability (%)
Gefitinib [μM]
H
HCC1806
Lapatinib resistant cells
Parental cells
Cell viability (%)
Lapatinib [μM]
J
HCC1806 Lapatinib resistant cells
sh-Control
SGCE-sh#1
SGCE-sh#2
Cell viability (%)
Lapatinib [μM]
G
MDA-MB-231
Gefitinib resistant cells
Parental cells
Cell viability (%)
Gefitinib [μM]
I
MDA-MB-231
Lapatinib resistant cells
Parental cells
Cell viability (%)
Lapatinib [μM]
K
MDA-MB-231 Lapatinib resistant cells
sh-Control
SGCE-sh#1
SGCE-sh#2
Cell viability (%)
Lapatinib [μM]

Fig. S8

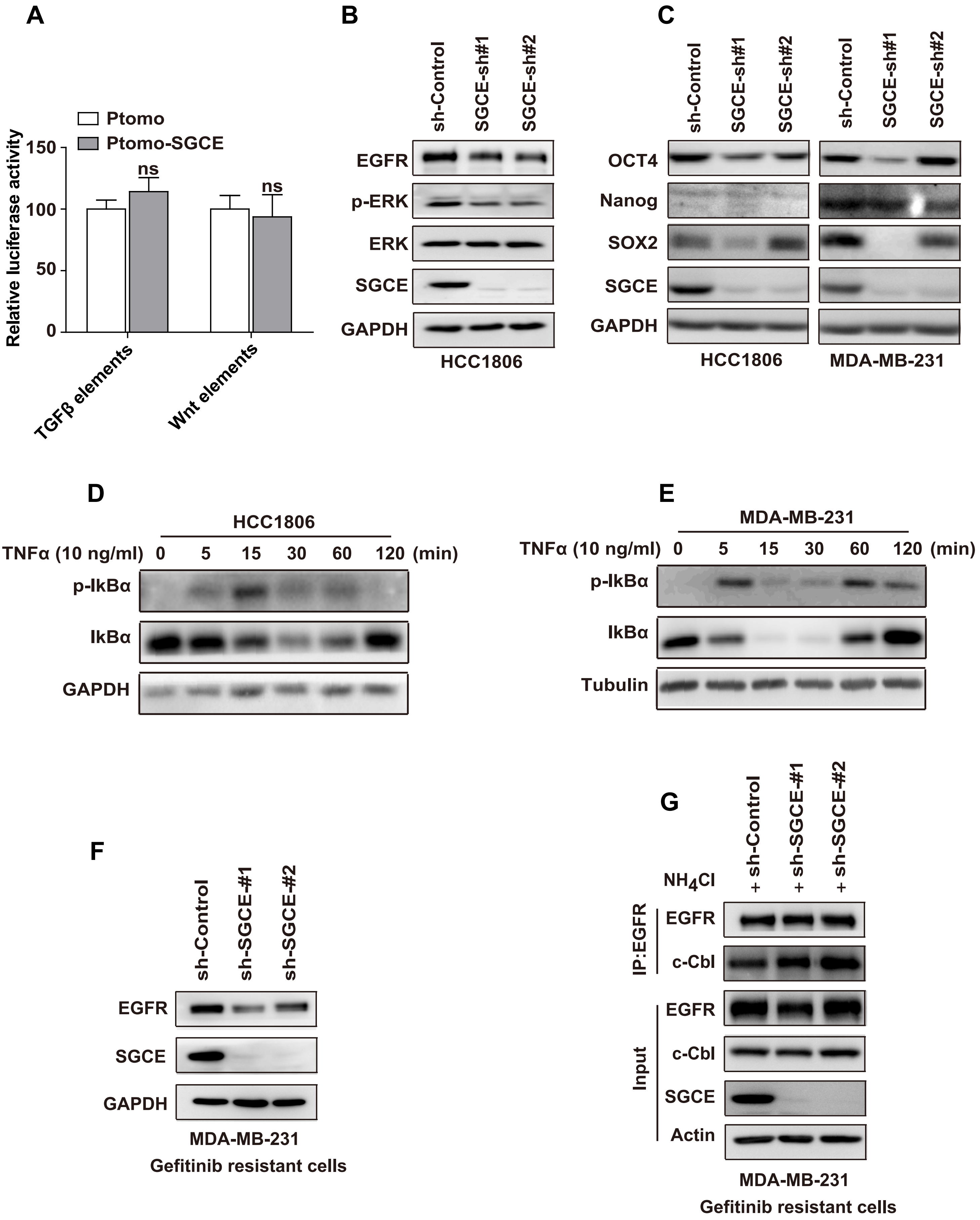
A
Ptomo
Ptomo-SGCE
Relative luciferase activity
150
100
50
0
ns
ns
TGFβ elements
Wnt elements
B
sh-Control
SGCE-sh#1
SGCE-sh#2
EGFR
p-ERK
ERK
SGCE
GAPDH
HCC1806
C
sh-Control
SGCE-sh#1
SGCE-sh#2
OCT4
Nanog
SOX2
SGCE
GAPDH
HCC1806
MDA-MB-231
D
HCC1806
TNFα (10 ng/ml) 0 5 15 30 60 120 (min)
p-IkBα
IkBα
GAPDH
E
MDA-MB-231
TNFα (10 ng/ml) 0 5 15 30 60 120 (min)
p-IkBα
IkBα
Tubulin
F
sh-Control
sh-SGCE-#1
sh-SGCE-#2
EGFR
SGCE
GAPDH
MDA-MB-231
Gefitinib resistant cells
G
NH4Cl
+ sh-Control
+ sh-SGCE-#1
+ sh-SGCE-#2
IP:EGFR
EGFR
c-Cbl
Input
EGFR
c-Cbl
SGCE
Actin
MDA-MB-231
Gefitinib resistant cells
